# Surface Molecular Markers for the Isolation of Viable Fibroblast Subpopulations in the Female Reproductive Tract: A Comprehensive Review

**DOI:** 10.3390/ijms26010233

**Published:** 2024-12-30

**Authors:** Krzysztof Łuszczyński, Michał Komorowski, Marta Soszyńska, Paulina Lewandowska, Robert Zdanowski, Monika Szafarowska, Paweł Kamiński, Marcin Niemcewicz, Jacek Malejczyk, Anna Lutyńska, Aneta Ścieżyńska

**Affiliations:** 1 Laboratory of Molecular Oncology and Innovative Therapies, Military Institute of Medicine National Research Institute, 128 Szaserów Street, 04-141 Warsaw, Poland; kluszczynski@wim.mil.pl (K.Ł.); rzdanowski@wim.mil.pl (R.Z.); alutynska@wim.mil.pl (A.L.); 2 Department of Histology and Embryology, Medical University of Warsaw, 02-004 Warsaw, Poland; michal.komorowski@wum.edu.pl (M.K.); marta.soszynska@wum.edu.pl (M.S.); plewandowska111@gmail.com (P.L.); jacek.malejczyk@wum.edu.pl (J.M.); 3 Department of Gynecology and Oncological Gynecology, Military Institute of Medicine, 128 Szaserów Street, 04-141 Warsaw, Poland; mszafarowska@wim.mil.pl (M.S.); pkaminski@wim.mil.pl (P.K.); 4 Biohazard Prevention Centre, Faculty of Biology and Environmental Protection, University of Lodz, 68 Narutowicza Street, 90-136 Lodz, Poland; marcin.niemcewicz@biol.uni.lodz.pl

**Keywords:** fibroblasts, subpopulations, gynecology, endometriosis, uterus, female reproductive track, FACS, scRNA-seq, surface markers

## Abstract

Advancements in single-cell analyzis technologies, particularly single-cell RNA sequencing (scRNA-seq) and Fluorescence-Activated Cell Sorting (FACS), have enabled the analyzis of cellular diversity by providing resolutions that were not available previously. These methods enable the simultaneous analyzis of thousands of individual transcriptomes, facilitating the classification of cells into distinct subpopulations, based on transcriptomic differences, adding a new level of complexity to biomolecular and medical research. Fibroblasts, despite being one of the most abundant cell types in the human body and forming the structural backbone of tissues and organs, remained poorly characterized for a long time. This is largely due to the high morphological similarity between different types of fibroblasts and the lack of specific markers to identify distinct subpopulations. Once thought to be cells responsible solely for the synthesis of extracellular matrix (ECM) components, fibroblasts are now recognized as active participants in diverse physiological processes, including inflammation and antimicrobial responses. However, defining the molecular profile of fibroblast subpopulations remains a significant challenge. In this comprehensive review, which is based on over two thousand research articles, we focus on the identification and characterization of fibroblast subpopulations and their specific surface markers, with an emphasis on their potential as molecular targets for selective cell isolation. By analyzing surface markers, alongside intra- and extracellular protein profiles, we identified multiple fibroblast subtypes within the female reproductive system. These subtypes exhibit distinct molecular signatures and functional attributes, shaped by their anatomical localization and the surrounding physiological or pathological conditions. Our findings underscore the heterogeneity of fibroblasts and their diverse roles in various biological contexts. This improved understanding of fibroblast subpopulations paves the way for innovative diagnostic and therapeutic strategies, offering the potential for precision targeting of specific fibroblast subsets in clinical applications.

## 1. Introduction

Fibroblasts are one of the most abundant cells in the human body and, as a pivotal part of the stroma, they form the fundamental structure of numerous tissues and organs [1]. Previously, fibroblasts were primarily recognized as cells responsible solely for the production and maintenance of extracellular matrix (ECM)-rich connective tissues, such as the endometrial stroma and the reticular dermis of the skin. However, in light of recent findings, it has become clear that fibroblasts not only provide support and positional information to adjacent cells through mechanical interactions, but are also involved in numerous other processes. They secrete various soluble mediators, including pro-fibrotic cytokines, i.e., TGFβ [2], and pro-inflammatory chemokines, i.e., CCL2 and CCL19 [3]. Moreover, cancer-associated fibroblasts, in order to facilitate tumor progression, could secrete several growth factors and metabolites [4]. Furthermore, by triggering the host immune system, they serve as key regulatory factors during inflammation and infections [5,6].

Fibroblasts are well-known for their remarkable plasticity [7]. In mature tissues, they typically remain in a quiescent state, becoming activated only in response to tissue repair or structural alterations. During development and repair processes, fibroblasts undergo transcriptional changes similar to cellular differentiation, suggesting a lineage hierarchy [8]. Quiescent fibroblasts can rapidly divide into ECM-secreting fibroblasts and other mesenchymal lineages, such as adipocytes, in response to injury [9,10]. Signalling and physical factors can induce the conversion of quiescent fibroblasts into myofibroblasts, which facilitate biomechanical remodeling and tissue contraction by generating traction forces on the newly formed extracellular matrix (ECM) [8].

Myofibroblasts not only drive tissue contraction, but also transform the surrounding environment by modulating immune cell functions and phagocytosing dead cells [11,12,13]. While crucial for acute injury repair, the chronic activation of myofibroblasts can lead to excessive ECM production, causing scarring, fibrosis, and potentially aiding tumorigenesis. This abnormal ECM disrupts tissue architecture, leading to organ dysfunction and contributing to nearly 50% of the death rate in developed countries [8].

Even though fibroblasts are primarily linked to human skin, their role in the physiology of the female reproductive system is just as important. Endometrial stromal fibroblasts (eSFs) are vital for endometrial functions. In response to estradiol and progesterone stimulation, they facilitate pregnancy and maintain tissue homeostasis [14]. In endometriosis, an estrogen-dependent chronic inflammatory disorder, eSFs develop resistance to progesterone, which contributes to infertility and poor pregnancy outcomes observed in affected women [15]. Moreover, eSFs are involved in a plethora of processes, such as decidualization, in which fibroblasts are transformed into decidual stromal cells (DSCs) and exhibit a molecular profile indicative of epithelial–mesenchymal transformation (EMT), which is crucial for blastocyst implantation [16,17]. Furthermore, fibroblasts take part in post-menstruation endometrium regeneration and healing after gynecological operations [18]. It has been shown that disruptions to these processes can lead to implantation failure and miscarriages [19]. Alterations in collagen metabolism and fibroblast function could lead to weakening of the pelvic floor and, as a result, to pelvic organ prolapse [20].

Female reproductive system cancers remain one of the leading causes of death in developed countries. A recent breakthrough in cancer research was the discovery that the severity, growth potential, and ability of a tumor to metastasize are influenced not only by tumor cells, but also by genetically normal stromal cells encircling the tumor [21]. Among these stromal cells, cancer-associated fibroblasts (CAFs), which have been described in regard to many gynecological cancers, such as cervical cancer, endometrial cancer, and ovarian cancer, play a significant role [22]. Cancer-associated fibroblasts (CAFs) are spindle-shaped cells that surround tumors and lack the characteristic markers of endothelial, epithelial, or leukocyte lineages. They are distinguished from normal fibroblasts by the expression of specific markers, such as Alpha-Smooth Muscle Actin (α-SMA) and Fibroblast-Specific Protein 1 (FSP1). Through paracrine signalling, the secretion of extracellular matrix (ECM) remodeling proteins, and the establishment of an immunosuppressive tumor microenvironment, CAFs play a crucial role in promoting cancer progression and facilitating immune evasion [23].

However, despite their clinical significance, fibroblast populations have for a long time been one of the least characterized cell types [24]. The recent advances in single-cell analyzis methods, particularly single-cell RNA sequencing (scRNA-seq), which have enabled the analyzis of thousands of individual cell transcriptomes, revealing numerous genes associated with distinct fibroblast lineages [25] and Fluorescence-Activated Cell Sorting (FACS), have enabled the analyzis of fibroblast subtypes at the cellular level [26]. However, due to the high morphological similarity between different types of fibroblasts, the variety of numerous fibroblast subpopulations, and the lack of specific surface markers, the sorting and in-depth analyzis of fibroblasts are almost impossible. Consequently, the majority of the research in this area is focused on the analyzis of bulk fibroblast populations, isolated from tissues using enzymatic methods. Moreover, studies identifying novel fibroblast subtypes often lack follow-up functional analyzes of viable, isolated subpopulations. Therefore, it is crucial to precisely characterize novel fibroblast subtypes, not only through the use of bioinformatic approaches, such as clustering algorithms, but also through the use of molecular studies, using carefully selected gene panels and surface protein markers. Functional studies are essential to elucidate the specific roles and mechanisms of fibroblast subtypes in tissue homeostasis and disease. Understanding the functional implications can reveal new therapeutic targets and enhance the development of novel precision medicine diagnostic and therapeutic strategies. Such detailed analyzis will significantly advance our knowledge of fibroblast biology and their involvement in various pathologies.

In order to address this knowledge gap, we present a comprehensive review of over two thousand papers, detailing the surface markers across various fibroblast populations present in the female reproductive system that can be used for cell sorting in future functional research.

## 2. Results

**Figure 1:**
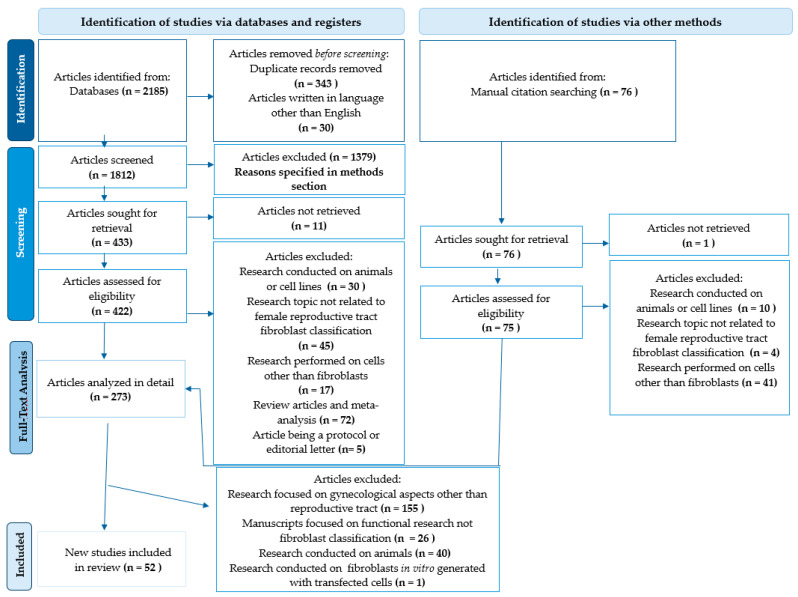
Flowchart of the selection process for the examined research publications.

A flowchart illustrating the selection process for the literature from the last ten years is depicted in Figure 1. Initially more than two thousand articles were chosen for the analyzis. After applying the inclusion criteria, the articles that did not meet the required standards, specifically those not published in English or involving studies conducted on cell lines and/or animal models, were excluded. As a result, 455 research papers were deemed to be eligible and were, subsequently, screened. Furthermore, irrelevant articles in regard to the research topic were excluded, leading to the final selection of 52 articles for detailed analyzis.

Table 1 shows the data obtained via single-cell sequencing of fibroblast subpopulations in normal non-pathologic tissues. Alongside endometrial and myometrial fibroblasts, pregnancy-related fibroblast subtypes present in placental villi were characterized. These studies identified genes encoding 14 unique surface markers: Adhesion G Protein-Coupled Receptor L4 (*ADGRL4*), Platelet-Derived Growth Factor Receptor Alpha (CD140a), Cadherin-11 (*CDH11*), Protein Delta Homolog 1 (*DLK1*), Estrogen Receptor (*ER1*), Heat Shock Protein HSP90-alpha (*HSP90AA1*), Neprilysin (CD10), Peripheral Myelin Protein 22 (*PMP22*), Prolactin Receptor (*PRLR*), Monocarboxylate Transporter 10 (*SLC16A10*), Sphingosine-1-Phosphate Phosphatase 1 (*SPP1*), Tissue Factor Pathway Inhibitor (*TFPI*), Transferrin Receptor Protein 1 (*TFRC*), and Thrombospondin-1 (*THBS1*). Furthermore, the studies also identified genes encoding 63 unique non-surface proteins, among which 27 were intracellular (red), 17 were extracellular matrix markers (green), and 19 were secreted proteins (blue).

Table 2 presents the results of the single-cell sequencing analyzes of fibroblast subtypes in endometriosis, including adenomyosis and uterine leiomyomas, the most prevalent benign gynecological disorders [33]. Apart from normal fibroblasts, various subtypes of myofibroblasts involved in the remodeling of the ECM were described. These analyzes identified genes encoding 28 unique surface markers, i.e., Platelet-Derived Growth Factor Receptor Alpha and Beta (CD140a and CD140b), Estrogen Receptor (*ESR1*), Neprilysin (CD10), Thy-1 Membrane Glycoprotein (CD90), Receptor Activity-Modifying Protein 1 (*RAMP1*), Sodium/Potassium-Transporting ATPase Subunit Beta-1 (*ATP1B1*), Bone Marrow Stromal Antigen 2 (*BST2*), Lysosome-Associated Membrane Glycoprotein 5 (*LAMP5*), Transmembrane Protein 119 (*TMEM119*), the Fibroblast Activation Protein (*FAP*), Voltage-Dependent L-type Calcium Channel Subunit Alpha-1C (*CACNA1C*), and Protein S100-A10 (*S100A10*). Moreover, genes encoding 43 intracellular markers (red), 17 extracellular matrix proteins (green), and 28 secreted proteins (blue) were described.

Table 3 shows the data obtained from single-cell analyzes of fibroblast subpopulations in ovarian cancers. The results are divided into Table 3—part (a), describing fibroblast subtypes in high-grade serous ovarian cancer (HGSOC), and Table 3—part (b), presenting fibroblast populations in epithelial ovarian cancer.

In both diseases, a plethora of disease-specific cancer-associated fibroblasts were found. However, no common surface markers were specified. In HGSOC fibroblast subtypes, 19 unique genes encoding the surface proteins were identified. Furthermore, genes encoding 36 unique intracellular markers (red), 10 extracellular matrix proteins (green), and 36 secreted proteins (blue) were described. In epithelial ovarian cancer, individual fibroblast populations were characterized by genes encoding 13 unique surface markers. Additionally, the studies found genes encoding 14 intracellular proteins (red), 8 extracellular matrix proteins (green), and 19 secreted proteins (blue).

Table 4 presents the results of the single-cell sequencing analyzes of fibroblast subtypes in endometrial cancer and immature ovarian teratoma. These studies found that highly active metabolic cancer-associated fibroblasts in endometrial cancer are characterized by the expression of the gene encoding Solute Carrier Family 2; Facilitated Glucose Transporter Member 1 (*SLC2A1*), a membrane protein involved in glucose uptake; Beta-1,4-Galactosyltransferase 1 (*B4GALT1*); and GTP-binding protein GEM (*GEM*). Furthermore, fibroblast subtypes in endometrial cancer were defined by genes encoding 20 intracellular proteins (red), 5 extracellular matrix proteins (green), and 10 secreted proteins (blue). Fibroblast subpopulations in immature ovarian teratoma were characterized by the expression of genes encoding three extracellular matrix proteins (green) and two secreted proteins (blue).

Table 5 shows the data obtained via single-cell sequencing of fibroblasts subpopulations in cervical cancer. These analyzes revealed genes encoding 46 unique surface markers. Moreover, the included studies described genes encoding 118 non-surface proteins, 55 were intracellular proteins (red), 17 were extracellular matrix proteins (green), and 46 were secreted proteins (blue). The analyzed research articles described a plethora of unique subpopulations of cancer-associated fibroblasts, which differ from those involved in the pathogenesis of ovarian cancer. Interestingly, one research paper described a subtype involved in the process of epithelial–mesenchymal transformation, characterized by the expression of genes encoding Desmocollin-3 (*DSC3*), Integrin Alpha-6 (*ITGA6*), Desmoplakin (*DSP*), and Cadherin-3 (*CDH3*), which correlates with recent findings describing the crucial role of CAFs in EMT in the cancer microenvironment [55].

Table 6 describes the publications that analyze various subpopulations of gynecology-associated fibroblasts using FACS or other antibody-based methods. The majority of the research has been performed on either healthy tissues or samples from patients with benign gynecological disorders. Most fibroblast populations were evaluated based on the expression of the surface markers CD73, CD90, CD105, CD140a, and CD140b, in various combinations. Consequently, the most abundant subtype of endometrial stromal fibroblasts present in both healthy and disease-affected tissue was characterized by the expression of genes encoding CD73, CD90, CD105, and CD140b. Fibroblasts from the placenta were also characterized. The table also includes negative surface markers, such as Receptor-Type Tyrosine-Protein Phosphatase C (CD45), the marker of hematopoietic cells, Cell Surface Glycoprotein MUC18 (CD146), the marker of endothelial cells in the vascular system, Hematopoietic Progenitor Cell Antigen CD34 (CD34), the marker for identifying stem cells, Platelet Endothelial Cell Adhesion Molecule (CD31), the marker for identifying leukocyte subsets, and Epithelial Cell Adhesion Molecule (CD326), the marker for identifying epithelial cells and epithelia-derived neoplasms.

## 3. Discussion

The female reproductive tract is believed to contain various fibroblast subpopulations that participate in a wide range of physiological processes. These subpopulations, along with the growing interest in precision and personalized medicine, represent promising therapeutic targets. The isolation and analyzis of these fibroblast subpopulations, although challenging due to the difficulty in identifying genes uniquely expressed in specific regions of the female reproductive tract but absent in other cell types, is crucial for understanding their specific role in inflammation, tissue repair, fibrosis, and malignancies. Fibroblasts are undoubtedly one of the most difficult cell types to classify, largely due to their high adaptability, the morphological similarity between different types of fibroblasts, the absence of distinct markers, and the limitations in terms of traditional research techniques, particularly Fluorescence-Activated Cell Sorting (FACS) of enzymatically digested tissues. Moreover, fibroblasts can exhibit significant variability within the same tissue, likely influenced by changes in the microenvironment.

The search for a universal set of pan-fibroblast markers, enabling selective and efficient cellular isolation, has continued for a long time and many molecules have been proposed as promising molecular targets, but all of them have distinct flaws. Small leucine-rich proteoglycans, such as Decorin (DCN) and Lumican (LUM), have been identified as highly specific pan-fibroblast markers [76]. However, they are unsuitable for viable cellular sorting applications. Due to their secretion following synthesis and the absence of a membrane-bound form, these molecules cannot be labeled in live cells for use in FACS analyzis. Although they are produced intracellularly, selective staining would necessitate cell permeabilization, which results in irreversible cellular damage and apoptosis [77]. Vimentin [78] is another marker with a similar issue, due to its mainly intracellular subcellular localization. Furthermore, it is also found in epithelial cells and macrophages [79]; therefore, it is not selective enough for sorting applications. Similarly, Fibroblast Specific Protein 1 (FSP1)/S100A4 [80] another promising at first marker not only exists mostly in the intracellular form but it is also present mostly in activated fibroblasts therefore not representing the entire cellular population. Over the years several membrane proteins such as Thy-1 (CD90) [81], Platelet-Derived Growth Factor Receptor Alpha (PDGFR-α; CD140a) [82], and the Fibroblast Activation Protein (FAP) [83] have been proposed as molecular targets and, although they were promising in the beginning, all of them have selectivity issues due to their expression in regard to several non-fibroblast cell types. For instance, Thy-1 is broadly expressed across various endothelial cells and mesenchymal stem cells. In contrast, FAPs are predominantly associated with activated fibroblasts and cancer-associated fibroblasts, rendering them less representative of the overall fibroblast population, and CD140a lacks specificity in regard to fibroblasts.

Various scientific methodologies have been implemented to analyze fibroblast subtypes. While the majority of studies are focused on single-cell RNA sequencing analyzes (scRNA-seq) and further bioinformatic-based divisions into specific subpopulations, Fluorescence-Activated Cell Sorting (FACS) of viable cells and combined approaches have also been implemented. However, these studies are very limited [84,85].

### 3.1. Fibroblast Markers Identified During Single-Cell RNA Sequencing (ScRNA-Seq)

In publications describing scRNA-seq results, the majority of researchers were focused on genes encoding intracellular proteins and describing fibroblasts’ secretory profiles (Table 1, Table 2, Table 3, Table 4 and Table 5). Therefore, due to the variety of marker combinations used and numerous subpopulations named, often without detailed, descriptive information, it is challenging and time consuming to re-analyze the data. In both healthy and pathological tissue, fibroblasts were divided based on their location and function.

The majority of fibroblast subpopulations derived from healthy endometrial tissue (Table 1, Figure 1) can be distinguished by the expression of the genes encoding hormone receptors [31], namely Estrogen Receptor 1 (ESR1), a fraction of which after palmitoylation binds to the cellular membrane [86] and the intracellular Progesterone Receptor (PGR). Therefore, hormonal stimulation could explain the structural and functional transformation of fibroblasts during the menstrual cycle [87]. Furthermore, endometrial stromal fibroblasts (eSFs) could also be distinguished and, subsequently, sorted from the pan-fibroblast population based on the expression of numerous genes encoding unique surface markers. These include Neprilysin (NEP, CD10), an integral membrane-bound metallopeptidase involved in the degradation of enkephalins and tachykinins, which regulates follicle maturation, ovulation, and ovarian blood flow, and is suggested to play a role in maternal–fetal interactions [88]; Platelet-Derived Growth Factor Receptor A (PDGFRA, CD140a), a tyrosine kinase receptor and potent activator of fibroblast proliferation and survival [89]; Adhesion G Protein-Coupled Receptor L4 (ADGRL4), a G-protein coupled receptor shown to promote angiogenesis and regulate migration and proliferation, whose overexpression facilitates the growth of several types of cancer [90]; Thrombospondin (THBS1), an adhesive glycoprotein involved in cell-to-cell and cell-to-matrix interactions, which has an important role in healing and wound repair, where its overexpression leads to delayed wound healing [91]; and Heat Shock Protein HSP90-alpha (HSP90AA1), a molecular chaperone that promotes protein folding and counteracts their aggregation [92]. The secretory profile of eSFs, comprising mostly of extracellular matrix-forming proteins, such as collagens and remodeling enzymes like metalloproteinases, highlights their significant role in the remodeling and rebuilding of the endometrial stroma during the menstrual cycle.

Interestingly, a unique subpopulation with potential involvement in uterine development was identified in placental villi samples [32]. While expressing genes encoding universal fibroblast markers, such as Decorin (DCN) and Collagen Type I Alpha 1 (COL1A1), were also characterized by the expression of unique genes encoding membranous proteins involved in epithelial–mesenchymal transformation, namely Cadherin-11 (CDH11) and transport proteins, such as Transferrin Receptor Protein 1 (TFRC, CD71) and Monocarboxylate Transporter 10 (SLC16A10). Moreover, it expressed genes encoding hormone receptors, such as the Prolactin Receptor (PRLR). This suggests that it could have numerous functions in placental development and in providing the developing fetus with the required amino acids in response to hormonal signalling.

**Figure 2:**
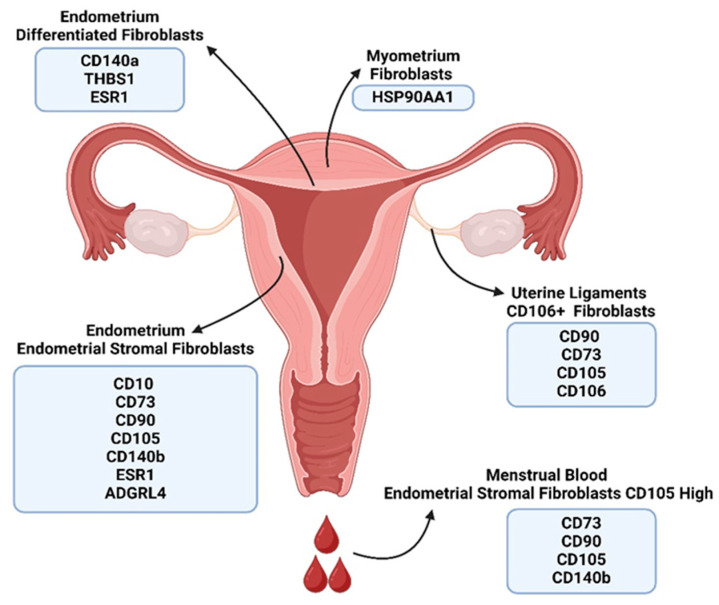
A graphical summary of various fibroblast subpopulations derived from healthy tissue and their corresponding surface markers. The major fibroblast subpopulations derived from the endometrium were endometrial stromal fibroblasts and differentiated fibroblasts, each characterized by a unique panel of surface markers. Moreover, fibroblasts with a similar profile to endometrial stromal fibroblasts were isolated from menstrual blood. Additionally, two distinct subtypes were isolated from myometrium and uterine ligaments. Created in BioRender.

In regard to endometriosis, one of the most common benign gynecological diseases, numerous unique surface and non-surface fibroblast markers have been identified (Table 2, Figure 2). However, very little similarity has been found among the subpopulations described across different studies. In endometriosis-derived tissue samples, both endometrial stromal fibroblasts (eSFs), which exhibit a gene expression profile similar to fibroblasts in healthy tissue, and unique endometriosis-specific fibroblasts (ESpecFs), have been described. ESpecFs express genes encoding surface proteins involved in gap junction formation, such as Gap Junction Protein Alpha 4 (GJA4), and genes protecting against FAS- or TNF-alpha-mediated apoptosis, such as Immediate Early Response 3 (IER3), as well as the intracellular c-FOS proto-oncogene. These fibroblasts display combined characteristics of both myofibroblasts, with a high level of expression of genes encoding extracellular matrix (ECM) components and remodeling proteins, such as collagens, metalloproteinases, and protease inhibitors, and inflammatory fibroblasts, expressing genes encoding pro-inflammatory chemokines, such as CXCL2, CXCL8, and C7. Additionally, they are involved in steroid hormone metabolism via the Steroidogenic Acute Regulatory Protein (*STAR*) gene, whose expression is closely associated with endometriosis [93].

**Figure 3:**
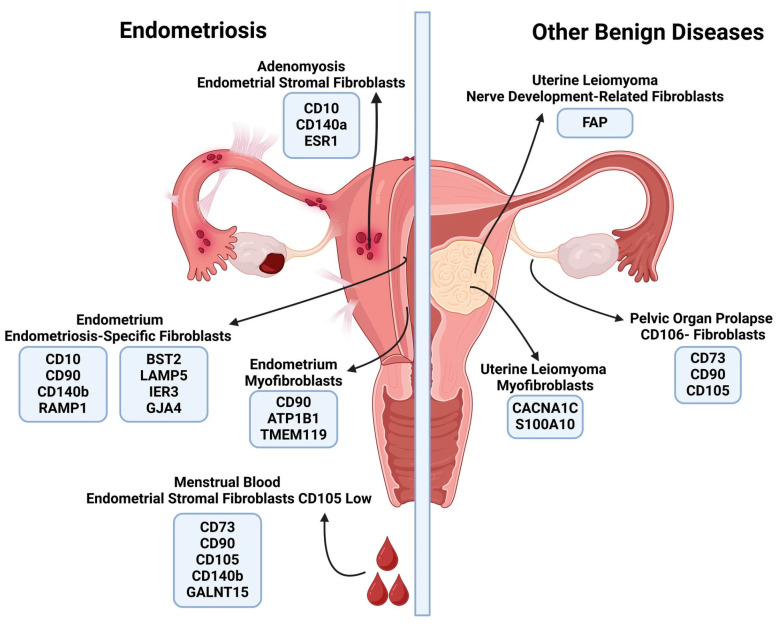
A graphical summary of various fibroblast subpopulations derived from benign gynecological diseases and their corresponding surface markers. The majority of research was conducted on fibroblasts derived from endometriosis tissue. Endometriosis-derived fibroblasts possess a surface marker profile similar to fibroblasts derived from healthy tissue, with a few unique surface markers. Other studied diseases were uterine leiomyoma and pelvic organ prolapse. A couple of unique fibroblast subtypes were described in regard to both of these diseases. Created in BioRender.

Uterine leiomyoma is the most common benign tumor found in the female reproductive tract. Fibroblasts forming extracellular matrix components of fibroids (Table 2, Figure 3) possess myofibroblast characteristics, with a high level of expression of ECM remodeling genes, i.e., *MYH11*, *MYL9*, or *DCN* and, as a result of paracrine mechanisms, the secretion of soluble growth factors are involved in the promotion of tumor growth [94]. The aforementioned fibroblasts could be characterized and sorted by genes expressing three distinct surface markers: the Fibroblast Activation Protein (FAP), a classic marker of activated fibroblasts; S100 Calcium-Binding Protein A10 (S100A10), a membrane protein involved in the promotion of cell growth, migration, and ECM remodeling [95]; and Voltage-Dependent L-Type Calcium Channel Subunit Alpha-1C (CACNA1C) [38]. Interestingly, Li et al. described that in tendons, increased calcium signalling is involved in fibrillogenesis and promotes the synthesis of numerous ECM components and remodeling proteins [96]. Therefore, a similar mechanism could also be proposed for matrix synthesis in uterine leiomyoma.

**Figure 4:**
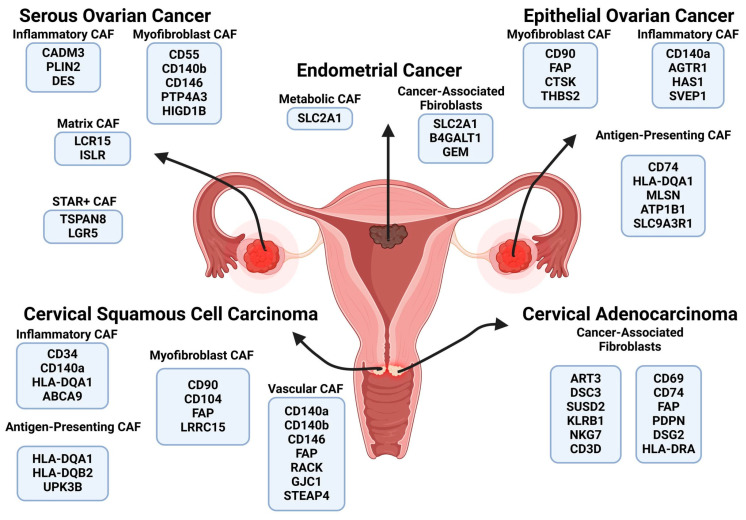
A graphical summary of numerous cancer-associated fibroblasts derived from gynecological cancers and their corresponding surface markers. Fibroblasts derived from cancer tissue show characteristics of cancer-associated fibroblasts (CAFs), with significant differences in the surface marker panel in comparison to fibroblasts derived from healthy tissue. The most prevalent cancer-associated fibroblast subtypes were myofibroblast CAFs, inflammatory CAFs, and antigen-presenting CAFs; among different tumors, they had similar, but not identical, expression profiles. Moreover, in regard to some tumors, unique cancer-associated fibroblast subpopulations were described. Created in BioRender.

Through the analyzis of fibroblast subpopulations in ovarian carcinomas, a broad spectrum of cellular heterogeneity was observed (Table 3, Figure 4). In accordance with the literature, Alpha-Smooth Muscle Actin (α-SMA), Integrin Beta-1 (CD29), the Fibroblast Activation Protein (FAP), Fibroblast-Specific Protein 1 (FSP1), and Platelet-Derived Growth Factor Beta Receptor (PDGFRβ), are fibroblast markers frequently associated with ovarian cancers [97,98]. However, during the relevant analyzis, these markers were only mentioned in a couple of papers. Loret et al. identified PDGFRβ among the cancer-associated fibroblasts in high-grade serous ovarian carcinoma (HGSOC) [40] and Mori et al. described FAP expression in a subset of fibroblasts associated with clear cell ovarian cancer [50]. Both of these subtypes shared characteristics of myofibroblast cancer-associated fibroblasts (mCAFs) because of the high level of expression of the genes encoding extracellular matrix building, i.e., COL4A1; ECM remodeling proteins, i.e., MMP11; and contractile proteins, i.e., TPPP3 and MYL9. The possible explanation is the high heterogeneity presented by ovarian carcinomas [99]. Ovarian cancers, particularly high-grade serous ovarian carcinoma, exhibit significant heterogeneity at both the molecular and cellular levels, which complicates the identification of a universal set of fibroblast activation-specific (FAS) surface markers. This heterogeneity is manifested in the diversity of genetic and epigenetic alterations, tumor microenvironment variations, and differential activation states of CAFs within and across patients. Consequently, the expression of FAS surface markers, such as the Fibroblast Activation Protein (*FAP*), Platelet-Derived Growth Factor Receptor (*PDGFR*), and C-X-C Chemokine Receptor Type 4 (*CXCR4*), can vary widely, reflecting the unique interactions between tumor cells and the surrounding stroma in each individual case [100].

A detailed analyzis of fibroblast subpopulations and their surface markers in HGSOC and epithelial ovarian cancer (EOC) revealed significant differences. In HGSOC, the major fibroblast subtypes were inflammatory cancer-associated fibroblasts (iCAFs), characterized by surface markers such as Cell Adhesion Molecule 3 (CADM3), Perilipin-2 (PLIN2), and Desmin (DES); myofibroblast CAFs (mCAFs), defined by markers including CD140b, Complement Decay-Accelerating Factor (CD55), Protein Tyrosine Phosphatase Type IVA 3 (PTP4A3), and HIG1 Domain Family Member 1B (HIGD1B); and stromal fibroblasts, characterized by Thy-1 (CD90), 5′-nucleotidase (CD73), and Endoglin (CD105). A portion of mCAFs also expressed CD146, a marker traditionally associated with endothelial cells and neoangiogenesis.

Additionally, three unique subpopulations have been described. The first group are matrix cancer-associated fibroblasts, characterized by the expression of genes encoding Leucine-Rich Repeat-Containing Protein 15 (LRC15) and Immunoglobulin Superfamily Containing Leucine-Rich Repeat Protein (ISLR), promoting immune cell migration via CXCL14 signalling [40]. The second novel subtype are STAR+ cancer-associated fibroblasts, mainly present in tumor lesions. They showed low expression levels of genes encoding classic CAF markers, such as *FAP* or *PDPN*, and instead had strong expression of stemness-associated genes encoding both membranous proteins, such as Tetraspanin-8 (*TSPAN8*) and Leucine-Rich Repeat-Containing G-Protein Coupled Receptor 5 (*LGR5*), as well as intracellular proteins, such as *STAR* and *ALDH1A1*. These cells, which are especially enriched after chemotherapy, were linked to improved prognoses and had a potential tumor-suppressing role [40]. Furthermore, a highly metabolically active metabolic cancer-associated fibroblast defined by the expression of SLC2A1, a membrane glucose transporter, was described.

On the other hand, in epithelial ovarian cancer, the main CAF subpopulations were myofibroblast CAFs characterized by FAP, CD90, Thrombospondin-2 (THBS2), and Cathepsin K (CTSK); inflammatory CAFs, defined by CD140a, Hyaluronan Synthase 1 (HAS1), Sushi, von Willebrand factor type A, EGF, pentraxin domain-containing protein 1 (SVEP1), and Type-1 Angiotensin II Receptor (AGTR1); and antigen-presenting CAFs (apCAFs), characterized by strong immunosuppressive properties, with their main surface markers being HLA-DQA1, HLA Class II Histocompatibility Antigen Gamma Chain (CD74), and Transient Receptor Potential Cation Channel Subfamily M Member 1 (MLSN) [50]. Moreover, apCAFs are the latest subtype of CAFs to be discovered, which through antigen stimulation of lymphocytes without co-stimulatory factors leads to their latency and apoptosis, therefore creating localized immunosuppression [101].

The analyzis of non-surface fibroblast markers in HGSOC and EOC revealed significant differences in their expression profiles. In HGSOC, the key findings include the prominence of extracellular matrix proteins, such as Alpha-1 Type I Collagen (COL1A1) and Collagen Type III, Alpha 1 (COL3A1), highlighting the critical role of extracellular matrix remodeling in HGSOC fibroblast biology. Additionally, secreted markers, mainly pro-inflammatory cytokines, i.e., IL6, CXCL10, and CXCL12, and extracellular matrix building proteins, i.e., Fibronectin (FN1), and ECM remodeling proteinases, i.e., MMP11, underscore the dynamic interplay between fibroblasts and their microenvironment. Conversely, Table 3—part (b) delineates fibroblast markers specific to epithelial ovarian cancer. Similar to HGSOC, there is a diverse range of intracellular, extracellular matrix-related, and secreted markers, suggesting a complex tumor–stroma interaction in EOC. Our analyzis identified shared markers among the fibroblasts from both carcinoma types, notably DCN, VIM, ACTA2, CXCL12/14, and COL1A1, which were prominent markers, characteristic of these cells. Among these shared markers, ACTA2 was the most prevalent, followed by COL1A1. However, the variability in the expression of the other markers between the two cancer types highlights the heterogeneity and distinct molecular profiles of fibroblasts associated with different ovarian cancer subtypes [102,103].

In immature ovarian teratoma, a rare benign tumor (Table 4), the fibroblast subpopulation resembled normal fibroblasts, but due to a lack of specific surface markers, their isolation and study remains challenging [52].

Endometrial cancer is one of the most common malignancies affecting women in developed countries, with a rapidly increasing incidence rate and a high disease-related economic burden [104]. Therefore, understanding its cellular composition could enable novel diagnostic and treatment options. Endometrial cancer-derived fibroblasts (Table 4, Figure 3) present the characteristics of myofibroblast cancer-associated fibroblasts, expressing genes encoding collagens and extracellular matrix remodeling proteases, or inflammatory cancer-associated fibroblasts, expressing genes encoding pro-inflammatory cytokines, i.e., CXCL12, CXCL14, and CCL11. The genes encoding surface markers, such as Beta-1,4-Galactosyltransferase 1 (*B4GALT1*), Solute Carrier Family 2, Facilitated Glucose Transporter Member 1 (*SLC2A1*), and GTP-binding protein GEM (*GEM*), a protein involved in numerous physiological processes, i.e., the suppression of cytoskeletal rearrangements and the inhibition of activity of Voltage-Dependent Calcium Channels [105], were highly overexpressed in endometrial cancer-derived CAFs. Moreover, their involvement in estrogen-responsive cancer proliferation and migratory molecular pathways make them promising clinical candidates [54]. Furthermore, SCL2A1 plays a crucial role in tumor glucose transport and metabolism [106,107], and B4GALT1, an N-glycan synthesis-related gene, facilitates N-glycosylation by catalyzing the transfer of β-1,4-linked galactose residues to acceptor sugar molecules and, which through N-linked glycosylation of PD-L1, assists cancer immune evasion [108]. Both of these genes encode membranous proteins, making them potential markers for isolating highly physiologically active cancer-associated fibroblasts.

Cervical cancer is the fourth most common malignancy among women. Despite the fact that in developed countries through the use of HPV vaccinations and lifestyle modifications its incidence is steadily decreasing, it is still prevalent in low-income regions [109]. Fibroblasts in cervical cancer (Table 5, Figure 4) are highly heterogeneous, with an expression profile similar to inflammatory, myofibroblast, or antigen-presenting cancer-associated fibroblasts, suggesting a complex cancer microenvironment with diverse interactions. In the analyzed studies, no universal surface markers have been found for inflammatory cancer-associated fibroblasts (iCAFs), although several promising candidate genes have been proposed [60,62,66] (Table 5). Those include Early Activation Antigen CD69 (CD69), Hematopoietic Progenitor Cell Antigen CD34 (CD34), and ATP-Binding Cassette Sub-Family A Member 9 (ABCA9). Despite their potential, none of these genes could be applied as a universal marker for iCAFs. Myofibroblast cancer-associated fibroblasts (mCAFs) are the most abundant subtype within cervical cancer stroma, exhibiting a characteristic secretion profile and a wide range of shared surface markers across various studies (Table 5). They can be defined by a panel of genes encoding well-known mCAF markers, such as THY-1 (CD90) [58] or the Fibroblast Activation Protein (*FAP*) [64]. Additionally unique surface proteins have been identified, including Integrin Alpha-1 (ITGA1), Receptor Activity-Modifying Protein 1 (RAMP1), the Prolactin Receptor (PRLR), COLEC12 collectin subfamily member 12 (COLEC12), Ras homolog gene family member B (RHOB), and Integrin Alpha-1 Leucine-Rich Repeat-Containing Protein 15 (*LRRC15)* [62]. Moreover, a subgroup of mCAFs was distinguished, namely vascular cancer-associated fibroblasts (vCAFs), by some research groups [65]. They are characterized by the expression of surface endothelial markers, namely Cell-Surface Glycoprotein MUC18 (CD146), and are associated with neoangionesis and vascular development [101]. The latest subpopulation to be described are apCAFs, which in cervical cancer are not defined by classical activation markers, i.e., the FAP, in comparison to other CAF subtypes; on the other hand, they express genes encoding uroplakin 3B (UPK3B) and MHC II complexes, i.e., HLA class II Histocompatibility Antigen Gamma Chain (CD74), *HLA-DQA1* or *HLA-DRB2* or a secreted protein, ADAMDEC1 (ADAM-Like Decysin 1).

### 3.2. Fibroblast Markers Identified Through Antibody-Based Techniques

A complementary approach to scRNA-seq for identifying and describing fibroblast subpopulations involves the application of antibody-based techniques, primarily through FACS. The majority of research (Table 6, Figure 1, Figure 2 and Figure 3) has been performed on either healthy tissue fragments or tissue derived from patients with benign gynecological conditions. In contrast to scRNA-seq-based studies, these investigations have been focused on isolating one or two of the most abundant fibroblast subtypes.

The first group of studies was focused on endometrial fibroblasts. Endometrial stromal fibroblasts (eSFs), derived from both healthy endometrium and endometriotic tissues, were characterized by a similar panel of surface markers. The most prominent markers include Thy-1 (CD90), a highly conserved protein anchored to the cell membrane via a glycosylphosphatidylinositol (GPI) anchor, which is involved in various physiological processes through the modulation of the WNT/β-catenin signaling pathway [81]; 5′-nucleotidase (CD73), an enzyme that converts extracellular adenosine monophosphate (AMP) into adenosine, thereby reducing local inflammation and promoting tissue repair [110]; Endoglin (CD105), a type I membrane glycoprotein and coreceptor of the TGF-β superfamily, which regulates angiogenesis and local immunosuppression through Prostaglandin E2 (PGE2)-mediated inhibition of cytotoxic T lymphocytes [111]; and Platelet-Derived Growth Factor Receptor B (CD140b). The results acquired from antibody-based studies on healthy tissue were focused on a different set of markers than scRNA-seq studies, therefore making a direct comparison between the described subpopulations is difficult. Additional studies are needed to fill this knowledge gap. Endometriosis-derived fibroblasts were characterized by lower levels of CD105, in comparison to cells derived from healthy tissue. Cells collected from menstrual efflux could be characterized by the same panel of surface markers as those collected via endometrial biopsy, suggesting that menstrual blood could serve as an accessible and cost-effective source of endometrial fibroblasts for further research [68]. Several markers of endometriosis-derived fibroblasts were shared between scRNA-seq and FACS studies, with CD90 and CD140b being the most prominent, while CD105 expression was absent in both methods. However, the expression and selectivity of numerous markers identified in next-generation sequencing studies should be confirmed at the protein level using FACS.

The second group of studies examined fibroblast subpopulations derived from extracellular matrix-rich tissues, such as uterine fibroids and the uterosacral ligament. In uterine fibroids, fibroblasts were characterized solely by CD90, which, being a very prevalent marker, makes their isolation and further classification difficult. Fibroblasts isolated from the uterosacral ligament exhibited a high level of similarity to endometrial stromal fibroblasts, with both subtypes lacking CD140b expression [73]. This similarity suggests shared physiological roles in maintaining tissue structure and responding to mechanical and hormonal signals in reproductive tissues. This alignment highlights potentially unique functionality of these fibroblast subtypes, such as specialized extracellular matrix remodeling or reduced proliferative activity, which may be critical for their roles in tissue homeostasis and adaptation within their respective environments. Additionally, a specific subpopulation could be distinguished by the expression of Vascular Cell Adhesion Protein 1 (CD106), a glycoprotein that plays a key role in leukocyte adhesion and transendothelial migration via its interaction with the α4β1 integrin [112]. This subtype was noted for its strong colony-forming capabilities and collagen production. Its prevalence was significantly reduced in pelvic organ prolapse-derived tissues, suggesting that it has a role in supporting tissue tension resistance. However, since FACS and scRNA-seq studies focus on different surface marker panels, additional studies using a pre-selected marker panel are needed for the direct comparison of these subpopulations.

The placenta is a highly heterogeneous organ, formed through complex interactions between fetal and maternal tissues. This heterogeneity is reflected in the results of the FACS studies. Boss et al. [75] described the presence of myofibroblasts characterized by CD90 and Dipeptidyl Peptidase 4 (CD26), a glycoprotein receptor essential for T-cell activation, and which is strongly associated with fibrosis and wound healing [113] in human placental tissue. In contrast, Riddell et al. [74] described a unique subpopulation characterized by the expression of the fibroblast marker TE-7 and the monocytic markers CD115 and CD14, which produced both pro-angiogenic growth factors and fibroblast growth factors. No surface markers were shared with cells described in the scRNA-seq studies (Table 1), indicating that more research is needed to fully understand the cellular composition of the placenta.

To date, the majority of fibroblast research has been performed on unsorted populations, which limits our knowledge of their diversity and functionality. Further research, particularly with the use of single-cell sequencing analyzis or FACS, should be focused on discovering new markers and methods to facilitate in-depth studies of isolated pure subtypes and to decipher their responses to different stimuli. In our research, we highlighted the most prevalent surface markers of the described and defined fibroblast subpopulations.

Following our analyzis, we selected markers based on their established roles in defining the identity of fibroblasts, their involvement in key biological processes, and their ability to differentiate between subpopulations across various conditions. For healthy tissues (Figure 2), we prioritized well-characterized markers that reflect baseline fibroblast functions like tissue support and repair. For endometriosis and other benign conditions (Figure 3), we chose markers known to vary in response to local inflammation, hormonal changes, and altered extracellular matrix dynamics. In characterizing cancer-associated fibroblasts (Figure 4), we focused on markers implicated in tumor progression, immune evasion, and metabolic support, ensuring that each chosen marker offered meaningful insights into both the cellular state and its impact on surrounding tissues. This approach ensured that our profiles not only distinguish different fibroblast subtypes, but also highlight the biological context in regard to their activities.

## 4. Materials and Methods

Selected literature (n = 2185) was retrieved from the National Center for Biotechnology Information Database (https://www.ncbi.nlm.nih.gov/ accessed on 13 December 2023 and revisited on 12 March 2024). The search terms were “Humans”, “Fibroblasts”, “Population”, “Sorting”, “Surface Marker”, “Heterogeneity”, “Discrimination”, “Single Cell”, “FACS”, “Subpopulation”, “Subtype”, “Phenotypes”, “Gynecology”, “Uterus”, “CD”, “Surface”, ”Human”, “Uterus”, “Ovary”, “Endometrium”, “Myometrium”, and “Endometriosis”, combined in regard to various modifications with the Boolean operators “AND” and “OR”. Additional information was obtained through hand searches of relevant articles (n = 76) not identified in the PubMed database, as well as from the references in the studied publications. Studies involving cell cultures and/or animals were omitted from the analyzis. The literature review was conducted from January to July 2024.

During the analyzis, the search results were initially collected, with duplicates and non-English manuscripts excluded. A preliminary screening was then performed based on the article titles and abstracts. Articles were excluded for the following reasons: studies on non-fibroblast cells (928), studies on non-human cells (171), a lack of fibroblast classification (267), and editorial letters or protocols (13). Additionally, reports were excluded if their full text was unavailable to the authors. Subsequently, 422 manuscripts from the PubMed database and 75 manuscripts obtained through manual searches were screened based on their full text. Following the selection process, 273 full-text reports were analyzed in detail, resulting in the inclusion of 52 articles, detailed in Table 1, Table 2, Table 3, Table 4, Table 5 and Table 6 of this manuscript. We analyzed the manuscripts by reviewing the key markers highlighted in the main text and the figures in each cited publication. If the details were limited, we examined the supplementary figures and experimental data, focusing on cellular markers. Markers were primarily chosen based on “avg_logFC” values; less frequent, but more specific, markers were occasionally included after group discussions, even if their “avg_logFC” was slightly lower than common markers, like collagen-encoding genes.

## 5. Conclusions

(1) Fibroblasts derived from the female reproductive tract are a highly heterogenous group, with each subtype possessing unique localizations, molecular profiles, and physiological functions characterized by a diverse panel of surface markers;
(2) The introduction of novel single-cell analyzis methods, mainly single-cell RNA sequencing (scRNA-seq) and Fluorescence-Activated Cell Sorting (FACS), has facilitated the selection and in-depth research on the biology of fibroblast subpopulations;
(3) Fibroblasts derived from healthy tissues display a distinct set of surface markers in comparison to fibroblast subtypes associated with benign gynecological disorders. These markers also significantly differ from those expressed by cancer-associated fibroblasts found in gynecological cancers;
(4) Our analyzis lists the most prevalent surface markers for each fibroblast subtype and therefore provides a foundation for new studies in this area;
(5) The specificity and expression levels of numerous markers discovered in scRNA-seq studies should be confirmed at the protein level using FACS and should be followed by functional studies;
(6) A thorough understanding of fibroblast functions in regard to both healthy and diseased states, particularly in relation to deleterious subpopulations, could facilitate their use as potential therapeutic targets for future biological drug development.

## Figures and Tables

**Table 1:** Main positive surface and non-surface markers of fibroblast subpopulations obtained from single-cell sequencing analyzes of normal tissue. The intracellular markers are labeled in red, the extracellular matrix markers are labeled in green, and the secreted markers are labeled in blue.

Publication	Medical Condition	Fibroblast Population	Main Positive Surface Markers					Main Positive Non-Surface Markers
[27]	Endometrium	Endometrial Stromal Fibroblasts	-					CFL1, ACTA2, DCN, COL6A1, LUM, COL5A1, COL6A3
[28]	Endometrium	Endometrial Stromal Fibroblasts	ADGRL4					COL5A1, LUM, COL6A3, DCN, IGFBP1
[29]	Endometrium	Secretory Endometrial Stromal Fibroblasts	-					SOCS3, CCN1, SCGB1D2, SCGB1D4, WFDC2, SCGB2A1
		Inflammatory Endometrial Stromal Fibroblasts	-					MT2A, SOD2, LMCD1, IL6, CXCL8
		SFRP4+ Endometrial Stromal Fibroblasts	-					RBP7, SFRP4, MMP11,
		DCN+ Endometrial Stromal Fibroblasts	-					HES1, BGN, CCDC80, C7, TIMP1, SPARCL1, MGP
[30]	Endometrium	C7+ Endometrial Stromal Fibroblasts	-					DCN, C7
		Non-Decidualized Endometrial Stromal Cells	-					CRABP2, MMP11
		Decidualized Endometrial Stromal Cells	-					FOXO1, CFD, IL15
[31]	Endometrium	Endometrial Stromal Fibroblasts	CD10	ESR1				PGR, COL1A1, LUM, MMP11, IGF1
		Endometrial Stromal Fibroblasts	CD10	ESR1				PGR
		Fibroblasts	-					WISP2, CCDC80, OGN, SERPINF1, GSN
		Differentiated Fibroblasts	CD140a	ESR1	THBS1			FOSB, EGR1, NR4A1, FOS, JUN
	Myometrium	ECM Remodeling Fibroblasts	-					CCN1, LUM, OGN, CTGF
		Fibroblasts	-					CRABP2, VCAN, MMP11, SFRP4
		Fibroblasts	-					CRABP2, ALDH1A2, RBP1, ECM1, SFRP4
		ECM Remodeling Fibroblasts	-					DCN, FBLN1, MGP, GSN
		Fibroblasts	HSP90AA1					FOS, EGR1
[32]	Placental Villi	Fibroblasts Involved in the Utero Development	CDH11	SLC16A10	PMP22	TFRC	PRLR	HSD17B2, SORBS2, TNNI3, RAB11FIP1, COL1A1, COL3A1, DCN, TGFBI
		Fibroblasts	SPP1					FTL, COL1A1, COL3A1, DCN, LAIR2
		ECM Remodeling Fibroblasts	DLK1	TFPI				MEST, CCDC68, NID2, COL6A3, COL1A1, COL3A1, DCN, F5

**Table 2:** Main positive surface and non-surface markers of fibroblast subpopulations obtained from single-cell sequencing analyzes of benign gynecological disorders. The intracellular markers are labeled in red, the extracellular matrix markers are labeled in green, and the secreted markers are labeled in blue.

Publication	Medical Condition	Fibroblast Population	Main Positive Surface Markers					Main Positive Non-Surface Markers
[31]	Adenomyosis–Endometriosis	Fibroblasts	-					ESR2
		Fibroblasts	-					COL3A1, MMP11
		ECM Remodeling Fibroblasts	-					STAT3, SMAD4, SPI, DCN, ASPN
		Fibroblasts	-					PGR, PAGE4, WIF1
		Endometrial Stromal Fibroblasts	CD140a	ESR1	CD10			RORB, HAND2, COL6A3
		Fibroblasts	-					PGR
[34]	Endometriosis	Fibroblasts	CD90	CD10	RAMP1			ATF3, VIM, DCN, COL1A1
		Myofibroblasts	CD90	ATP1B1				ACTA2, TAGLN, MYK, VIM, COL1A1, DCN, COL1A2
[35]	Endometriosis	Fibroblasts 1	BST2					C7
		Fibroblasts 2	BST2	LAMP5				HOPX, FNDC1, SFRP2
		Myofibroblasts 1	-					POSTN, COL6A1, SFRP1, FN1, PAMR1
		Myofibroblasts 2	-					POSTN, COL6A1, COL4A1, FN1, A2M
		Myofibroblasts 3	TMEM119					POSTN, COL6A1, TIMP3, TNC, FN1
[36]	Endometriosis	SC-FBs-1—Endometrial Stromal Fibroblasts (Control Tissue)	RAMP1	CD24				CKB, CRABP2, VIM, COL1A1, COL1A2, COL3A1, SFRP4
		SC-FBs-6—Endometrial Stromal Fibroblasts (Control Tissue)	CD24	DIO2				TXN, CRABP2, VIM, COL1A1, COL1A2, COL3A1, SFRP4
		SC-FBs-8—Endometrial Stromal Fibroblasts (Control Tissue)	-					PCLAF, TYMS, HMGB2, VIM, COL1A1, COL1A2, COL3A1
		SC-FBs-9—Endometrial Stromal Fibroblasts (Control Tissue)	-					CENPF, PTTG1, TOP2A, VIM, COL1A1, COL1A2, COL3A1
		SC-FBs-4—Stromal Fibroblasts	CD146	RGS16				ACTA2, ADIRF, RGS5, VIM, COL1A1, COL1A2, COL3A1
		SC-FBs-5—Stromal Fibroblasts	ESR1	PTCH1	DIO2			XIST, VIM, VCAN, COL5A1, COL1A1, COL1A2, COL3A1
		SC-FBs-7—Stromal Fibroblasts	GJA1					VIM, COL1A1, COL1A2, COL3A1, VCAN, IGF1
		SC-FBs-12—Stromal Fibroblasts	IGKC	IGHG3	IGHL3	IGHG4	IGHG1	S100A9, VIM, COL1A1, COL1A2, COL3A1, GNLY, VWF
		SC-FBs-13—Stromal Fibroblasts	NKG7	KLRB1				VIM, COL1A1, COL1A2, COL3A1, GNLY, SRGN, CCL4
		SC-FBs-2—Endometriosis Specific Fibroblasts	-					FOS, TCEAL4, VIM, COL1A1, COL1A2, COL3A1, C7
		SC-FBs-3—Endometriosis Specific Fibroblasts	IER3					STAR, VIM, COL1A1, COL1A2, COL3A1, CXCL8, CXCL2
		SC-FBs-10—Endometriosis Specific Fibroblasts	-					VIM, COL1A1, COL1A2, COL3A1, CTHRC1, TIMP1, SFRP2
		SC-FBs-11—Endometriosis Specific Fibroblasts	GJA4					CRYAB, VIM, COL4A1, COL1A1, COL1A2, COL3A1, CXCL8
[37]	Endometriosis	Endometrial Stromal Fibroblasts—Major Population (Control Tissue)	CD140b					ACTA2, CALD1, NUPR1, IGFBP5, CTGF, MMP11
		Endometrial Stromal Fibroblasts—Minor Population (Disease-Affected Tissue)	CD140b					CKS2, CST1, CST3, MMP1, MMP3, MMP10
[38]	Uterine Leiomyoma	Myofibroblasts	-					DES, MYH11, IER2, NR4A1, DCN, vWF, SERPINE1
		Nerve Development-Related Fibroblasts	FAP					ADH1B, COL6A3, LAMB1, CFD, SULF1
		ECM-Producing Fibroblasts	-					ADH1B, PI16, SFRP2, SULF1, SOD3
		Myofibroblasts	CACNA1C	S100A10				RGS5, MYL9, ACTG2, DCN, MFAP4, APOD

**Table 3:** Main positive surface and non-surface markers of fibroblast subpopulations obtained from single-cell sequencing analyzes of high-grade serous ovarian cancer. The intracellular markers are labeled in red, the extracellular matrix markers are labeled in green, and the secreted markers are labeled in blue.

Publication	Medical Condition	Fibroblast Population	Main Positive Surface Markers					Main Positive Non-Surface Markers
(**a**)								
[39]	High-Grade Serous Ovarian Cancer	Myofibroblasts	-					ACTA2, MYH11, TAGLN
		Stromal Fibroblasts CD73+, CD90+, CD105+	CD90		CD73	CD105		-
		Stromal Fibroblasts CD73+, CD105+			CD73	CD105		-
		Stromal Fibroblasts CD105+				CD105		-
		Myofibroblast Cancer-Associated Fibroblasts	-					PTHLH, COL3A1, TGFB3, FGF1, MMP11
[40]	High-Grade Serous Ovarian Cancer	Vascular/Myofibroblast Cancer-Associated Fibroblasts	CD140b		CD146	PTP4A3	HIGD1B	COX412, PPP1R14A, MEF2C, RGS5, COL4A1, MGP
		Inflammatory Cancer-Associated Fibroblasts	CADM3		PLIN2	DES		KRT7, CALB2, SERPINB2, IL6, CXCL12
		Matrix Cancer-Associated Fibroblasts	LRC15		ISLR			COL11A1, MFAP5, SFRP2, COL10A1, LOX, SFRP2
		STAR+ Cancer-Associated Fibroblasts	TSPAN8		LGR5			STAR, ALDH1A1, IGFBP5, C7, vWF
[41]	High-Grade Serous Ovarian Cancer	CAF	CD90		PDPN			DCN, C1Q1, C1QB, C1QC, CFB, CXCL1, CXCL2, CXCL10, CXCL12, IL6
[42]	High-Grade Serous Ovarian Cancer	CAF-FB0	-					ACTA2, VIM, WNT5A
		CAF-FB1	-					ACTA2, VIM, WNT5A
		CAF-FB2	-					ACTA2, VIM, WNT5A
		CAF-FB3	-					ACTA2, VIM, WNT5A
		CAF-FB4	-					ACTA2, VIM, WNT5A
		CAF-FB5	-					ACTA2, VIM, WNT5A
		CAF-FB6	-					ACTA2, VIM, WNT5A
		CAF-FB7	-					ACTA2, VIM, WNT5A
		CAF-FB8	-					ACTA2, VIM, WNT5A
[43]	High-Grade Serous Ovarian Cancer	INHBA+ Cancer-Associated Fibroblasts	-					ACTA2, INHBA
[44]	High-Grade Serous Ovarian Cancer	Myofibroblast Cancer-Associated Fibroblasts	-					IDO, COL1A1, DCN, CXCL10
		Myofibroblast Cancer-Associated Fibroblasts	-					COL1A1, DCN, MMP11
		Myofibroblast Cancer-Associated Fibroblasts	CD55					COL1A1, DCN, CXCL14
		Myofibroblast Cancer-Associated Fibroblasts	-					HK2, COL1A1, DCN, TGFB1, LGALS3
		Inflammatory Cancer-Associated Fibroblasts	-					HK2, COL1A1, DCN, IL6, IL1B
		Antigen-Presenting Cancer-Associated Fibroblasts	-					KRT19, HK2, COL1A1, DCN, CXCL12
[45]	High-Grade Serous Ovarian Cancer	Myofibroblasts	BCAM					TAGLN, ACTA2, MYL9, MYH11, PLN
		Fibroblasts 1	-					STAR, MEG3, DNAJB1, HSAP1B, DCN,
		Fibroblasts 2						RBP1, DCN, LUM, GSN
		Fibroblasts 3	RAMP1					TXNIP, CTSB, IGF1, PRSS23, CFD
		Fibroblasts 4	-					DNAJB1, DCN, CCL2, GSN
		Fibroblasts 5	-					COL1A1, COL1A2, COL3A1, SPARC, FN1
[46]	High-Grade Serous Ovarian Cancer	Matrix Cancer-Associated Fibroblasts						POSTN, CTHRC1, COL1A1, COL1A2, COL3A1, COL10A1, COL11A1, MMP11
		Inflammatory Cancer-Associated Fibroblasts						CFD, CCL11, CXCL12, CXCL14, APOD, C3
		Metabolic Cancer-Associated Fibroblasts	SLC2A1					HILPDA, BNIP3, NDRG1, ENO1, VEGFA, ADM
		Proliferative Cancer-Associated Fibroblasts						CENPF, NUSAP1, PTTG1, STMN1, TOP2A, TUBA1B
(**b**)								
[47]	Epithelial Ovarian Cancer	Cancer-Associated Fibroblasts	-					VIM, COL1A1
		ECM-Producing Cancer-Associated Fibroblasts	CD90					ACTA2, TWIST1, ZEB1, VIM, COL11A1
[48]	Epithelial Ovarian Cancer	Metastases-Supporting Cancer-Associated Fibroblasts						RGS5, COL4A1
		Cancer-Associated Fibroblasts	-					DCN, CTHRC1, FN1, RARRES2
[49]	Epithelial Ovarian Cancer	Metastatic Fibroblasts	-					ACTA2, ACTB, DCN, CXCL12, FSTL1, SFRP2, SFRP4, IGF1, CXCL14, ANGPTL4
		Primary Fibroblasts	-					ACTA2, ACTB, DCN, WNT5A
		Fibroblasts	-					ACTA2, ACTB, DCN, WFIKKN2
[50]	Epithelial Ovarian Cancer	Cancer-Associated Fibroblasts	-					VIM, COL1A1, COL1A2, DCN
		Myofibroblast Cancer-Associated Fibroblasts	CD90	FAP	THBS2	CTSK		TAGLN, MYL9, TPM1, CTHRC1, MMP11
		Inflammatory Cancer-Associated Fibroblasts	AGTR1	HAS1	SVEP1	CD140a		LMNA, TNXB, CXCL12, IL6, GSN
		Antigen-Presenting Cancer-Associated Fibroblasts	HLA-DQA1	CD74	MLSN	SLC9A3R1	ATP1B1	ARHGDIB, PTGIS, SAA2, CLU, SLPI
[51]	Ovarian Cancer	Cancer-Associated Fibroblasts	-					COL3A1, COL1A1, COL1A2, CTS3, MMP2
		Myofibroblast Cancer-Associated Fibroblasts	-					MYL9, MYLK, MYC

**Table 4:** Main positive surface and non-surface markers of fibroblast subpopulations obtained from single-cell sequencing analyzes of endometrial cancer and immature ovarian teratoma. The intracellular markers are labeled in red, the extracellular matrix markers are labeled in green, and the secreted markers are labeled in blue.

Publication	Medical Condition	Fibroblast Population	Main Positive Surface Markers			Main Positive Non-Surface Markers
[52]	Immature Ovarian Teratoma	Fibroblasts	-			COL1A1, COL1A2, COL3A1, MGP, PTGDS
[53]	Endometrial Cancer	Cancer-Associated Fibroblasts	-			ACTA2, COL1A1, SFRP4
[46]	Endometrial Cancer	Matrix Cancer-Associated Fibroblasts				POSTN, CTHRC1, COL1A1, COL1A2, COL3A1, COL10A1, MMP11
		Inflammatory Cancer-Associated Fibroblasts				CFD, CCL11, CXCL12, CXCL14, APOD, C3
		Metabolic Cancer-Associated Fibroblasts			SLC2A1	BNIP3, HILPDA, NDRG1, ENO1, VEGFA, ADM
		Proliferative Cancer-Associated Fibroblasts				CENPF, NUSAP1, PTTG1, STMN1, TOP2A, TUBA1B
[54]	Endometrial Cancer	Cancer-Associated Fibroblasts 1	GEM	B4GALT1		XBP1, UGCG, CCND1, TBX3, COL3A1, COL1A1
		Cancer-Associated Fibroblasts 2	GEM			TBX3, COL3A1, COL1A1
		Cancer-Associated Fibroblasts 3	GEM	B4GALT1	SLC2A1	XBP1, UGCG, FHL2, KRT19, PMAIP1, CCND1, TFAP2C
		Cancer-Associated Fibroblasts 4	GEM			XBP1, UGCG, FHL2, KRT19, PMAIP1, TBX3, COL3A1
		Cancer-Associated Fibroblasts 5	GEM			FHL2, TBX3, COL3A1, COL1A1
		Cancer-Associated Fibroblasts 6	GEM	B4GALT1		XBP1, UGCG, FHL2, KRT19, PMAIP1, CCND1, TFAP2C
		Cancer-Associated Fibroblasts 7	GEM			PMAIP1, TBX3, COL3A1, COL1A1

**Table 5:** Main positive surface and non-surface markers of fibroblast subpopulations obtained from single-cell sequencing analyzes of cervical cancer. The intracellular markers are labeled in red, the extracellular matrix markers are labeled in green, and the secreted markers are labeled in blue.

Publication	Medical Condition	Fibroblast Population	Main Positive Surface Markers					Main Positive Non-Surface Markers
[56]	Cervical Cancer	Inflammatory Cancer-Associated Fibroblasts	-					GSN, IL8, CXCL2, IL6, SOD3
		Myofibroblast Cancer-Associated Fibroblasts	RAMP1	PRLR	COLEC12	RHOB		ACTA2, MYH11, PGM2L1, SCARA3, CLU
[57]	Cervical Cancer	Cancer-Associated Fibroblasts	CD140a	FAP	PDGFC	PDPN		TWIST2, LUM, DCN, COL8A1, COL6A3
		Cancer-Associated Fibroblasts	CD146	CDH6	NOTCH3			RGS5, COL18A1, ANGPT2, PDGFA
		Epithelial–Mesenchymal Transformation Cancer-Associated Fibroblasts	DSC3	ITGA6	DSP	CDH3		KRT6A, KRT5, KRT19, CDKN2A, LY6K
		Cancer-Associated Fibroblasts	CD52					CXCL10, GNLY
		Cancer-Associated Fibroblasts						DPT, PTGDS, CCL21, CCL19, CXCL12
		Cancer-Associated Fibroblasts	CD74	HLA-DRB5	HLA-DQA1	HLA-DRB1		
[58]	Cervical Cancer	Myofibroblast Cancer-Associated Fibroblasts	CD90	ITGA1				POSTN, COL1A1, COL8A1, MMP11, FN1
		Matrix Cancer-Associated Fibroblasts	CD56					COL14A1, ADAMTS19
		Vascular Cancer-Associated Fibroblasts						LMCD1, EPAS1, SOD2, COL4A1, VEGFA
		Developmental Cancer-Associated Fibroblasts	PTCH1					DACH1, PAMR1
		Fibroblasts	PTPRC	MUC16				
[59]	Cervical Adenocarcinoma	Inflammatory Cancer-Associated Fibroblasts						CKB, DPT, CFD
		Myofibroblast Cancer-Associated Fibroblasts						SULF1, COL8A1, INHBA
[60]	Cervical Adenocarcinoma	Cancer-Associated Fibroblasts	FAP	PDPN				S100A4, COL1A2, DCN, LUM, COL10A1
		Cancer-Associated Fibroblasts	-					TAGLN, S100A4, VIM, DCN, PDGFA
		Cancer-Associated Fibroblasts	-					COL3A1, CFD, APOD, CXCL14, PTGDS
		Cancer-Associated Fibroblasts	HLA-DRA	CD74				AGR2, KRT8, RGS5, WFDC2, SLPI
		Cancer-Associated Fibroblasts	DSG2	ART3				STMN1, KRT19, KRT15, KANK4, ANLN
		Cancer-Associated Fibroblasts	DSC3	DSG2	SUSD2			KRT8, KRT14, MMP7, TGFB1, PTGDS
		Cancer-Associated Fibroblasts	KLRB1	CD69	NKG7	CD3D		GBP5, CCL5, CXCR4, CCL4, APOD
	Cervical Squamous Cell Carcinoma	Cancer-Associated Fibroblasts	LY6D					KRT6A, S100A8, S100A9, S100A2, KRT15
		Cancer-Associated Fibroblasts	CDH6	LY6D				ACTA2, S100A2, RGS5, S100A8, S100A9
		Cancer-Associated Fibroblasts	CD74	HLA-DRB5	HLA-DRA	FAP		RGS4, WARS, GBP5, EGR1, TAGLN
[61]	Cervical Squamous Cell Carcinoma	Immune-Associated Cancer-Associated Fibroblasts	CD140a	FAP				CNN1, TCF21, COL1A1, C7
		Vascular Cancer-Associated Fibroblasts	CD140a	FAP	RACK			ZC3H11A, SELENOM, COL1A1
		Stroma-Associated Cancer-Associated Fibroblasts	CD140a	FAP				MYEF2, CTGF, COL1A1, TNXB
[62]	Cervical Squamous Cell Carcinoma	Antigen-Presenting Cancer-Associated Fibroblasts	HLA-DQA1	HLA-DQB2	UPK3B			ADAMDEC1
		PI16+ Inflammatory Cancer-Associated Fibroblasts	HLA-DQA1	CD140a				PI16, CCL2, CXCL1, CXCL12
		ABCA9+ Inflammatory Cancer-Associated Fibroblasts	ABCA9					CCL2, CXCL1, CXCL12
		NPNT+ Myofibroblast Cancer-Associated Fibroblasts	CD90					CALD1, ACTA2, NPNT, GRP, ADAMDEC1
		LRRC15+ Myofibroblast Cancer-Associated Fibroblasts	LRRC15					CALD1, ACTA2, GRP, MMP11
		IGF1+ Myofibroblast Cancer-Associated Fibroblasts	CD90					IGF1, MMP11
		TOP2A+ Myofibroblast Cancer-Associated Fibroblasts	CD90	LRRC15				TOP2A, CALD1, ACTA2, MMP11, GRP
[63]	Cervical Squamous Cell Carcinoma	Myofibroblast Cancer-Associated Fibroblasts						ACTA2, S100A4, VIM, COL1A1, COL1A2
		Extracellular Cancer-Associated Fibroblasts						FBLN1, LUM, COL1A1, MMP11, MMP2
[64]	Cervical Squamous Cell Carcinoma	Myofibroblast Cancer-Associated Fibroblasts	FAP	CD104				ACTA2, POSTN
[65]	Cervical Squamous Cell Carcinoma	Matrix Fibroblasts	CD140a	CD140b	IER3			NFKBIA, ZFP36, LUM, DCN, CFD
		Matrix Fibroblasts	CD140a	CD140b				S100A6, LUM, DCN, IGFBP6, PI16
		Matrix Cancer-Associated Fibroblasts	CD140a	CD140b				LUM, DCN, VCAN, MMP11, WNT5A
		Vascular Cancer-Associated Fibroblasts		CD140b	CD146	GJC1	STEAP4	ACTA2, RGS5, NDUFA4L2, MYO1B, CCDC102B
		Vascular Cancer-Associated Fibroblasts		CD140b	CD146			ACTA2, RGS5, MYH11, MYL9, TAGLN
		Vascular Cancer-Associated Fibroblasts		CD140b	CD146			ACTA2, RGS5, MYH11, MYL9, MYLK
[66]	Cervical Squamous Cell Carcinoma	Fibroblasts EGR3+						CXCL1, CCL2, PTGDS
		Fibroblasts ID2+						ID2, PLCG2
		Fibroblasts NCAM1+						PTGDS, IGFBP5, CCN5
		Fibroblasts MKI67+						KI67
		Inflammatory Cancer-Associated Fibroblasts CD34+	CD34					ADH1B, C3, C7, A2M, CFD
		Myofibroblast Cancer-Associated Fibroblasts MMP11+	LRRC15					SULF1, POSTN, CTHRC1, MMP11, SFRP2
		Cancer-Associated Fibroblasts DES+						ACTA2, MYH11, DES, TAGLN, TPM1

**Table 6:** Main surface and non-surface markers of fibroblast subpopulations acquired through the use of FACS or other antibody-based analyzes. The intracellular markers are labeled in red, the extracellular matrix markers are labeled in green, and the secreted markers are labeled in blue.

Publication	Medical Condition	Fibroblast Population	Main Negative Surface Markers							Main Positive Surface Markers					Main Positive Non-Surface Markers
[67]	Endometrium	Endometrial Stromal Fibroblasts	CD45	-	CD146	CD326	-	-	-	-	-	-	CD140b	-	-
[68]	Healthy Endometrium (Menstrual Blood)	Endometrial Stromal Fibroblasts CD105 high	CD45	CD31	-	CD326	-	-	-	CD90	CD73	CD105	CD140b		ALDH1A1, TAGLN, TIMP1, TIMP2, WNT5A
	Endometriosis (Menstrual Blood)	Endometrial Stromal Fibroblasts CD105 low	CD45	CD31	-	CD326	-	-	-	CD90	CD73	CD105	CD140b	GALNT15	COL11A1, MMP7, WNT5A
[15]	Endometriosis	Endometrial Stromal Fibroblasts	CD45	-	CD146	CD326	-	-	-	-	-	-	CD140b	-	-
[69]	“Benign Conditions” (Endometrium)	CD90+ Fibroblasts	CD45	-	-	-	CD34	-	-	CD90	CD40	-	-	-	VIM
		CD90- Fibroblasts	CD45	-	-	-	CD34	-	CD90	-					VIM
[70]	Polycystic Ovary Syndrome (Endometrium)	Fibroblasts	CD45	-	CD146	-	-	-	-	-	-	-	CD140b	-	-
[71]	Uterine Fibroid	ALDH+/CD90+	CD45	CD31	-	-	-	-	-	CD90	-	-	-	-	ALDH, VIM
		ALDH-/CD90+ Bright	CD45	CD31	-	-	-	-	-	CD90	-	-	-	-	VIM
[72]	Uterine Fibroid	ALDH+/CD90+	CD45	CD31	-	-	-	-		CD90	-	-	-	-	ALDH
		ALDH-/CD90+ Bright	CD45	CD31	-	-	-	-		CD90	-	-	-	-	-
[73]	Pelvic Organ Prolapse (Uterosacral Ligament)	CD106- Fibroblasts	CD45	-	-	-	CD34	HLA-DR	CD106	CD90	CD73	CD105	-		ACTA2, S100A4, VIM, MMP1
		CD106+ Fibroblasts	CD45	-	-	-	CD34	HLA-DR	-	CD90	CD73	CD105	CD106	-	ACTA2, S100A4, VIM
[74]	Placental Samples	Fibrocyte-Like Placental Cells	CD45	CD31	CD146	-	-	-	PM-2K	CD14	CD115	VEGF-R2	TE-7	-	-
[75]	Placental Samples	Myofibroblasts	CD45	CD31	CD146	CD36	CD271	CD142	CD73	CD90	CD26	-	-	-	-

## Data Availability

All the relevant data are included within the manuscript. The raw data are available on request from the corresponding author.

## References

[1] Dick M.K., Miao J.H., Limaiem F. (2024). Histology, Fibroblast.

[2] Lemos D.R., Duffield J.S. (2018). Tissue-resident mesenchymal stromal cells: Implications for tissue-specific antifibrotic therapies. Sci. Transl. Med..

[3] He H., Suryawanshi H., Morozov P., Gay-Mimbrera J., Del Duca E., Kim H.J., Kameyama N., Estrada Y., Der E., Krueger J.G. (2020). Single-cell transcriptome analysis of human skin identifies novel fibroblast subpopulation and enrichment of immune subsets in atopic dermatitis. J. Allergy Clin. Immunol..

[4] Tian H., Wang W., Liang S., Ding J., Hua D. (2024). From darkness to light: Targeting CAFs as a new potential strategy for cancer treatment. Int. Immunopharmacol.

[5] Davidson S., Coles M., Thomas T., Kollias G., Ludewig B., Turley S., Brenner M., Buckley C.D. (2021). Fibroblasts as immune regulators in infection, inflammation and cancer. Nat. Rev. Immunol..

[6] Cavagnero K.J., Gallo R.L. (2022). Essential immune functions of fibroblasts in innate host defense. Front. Immunol..

[7] Morrison S.J., Spradling A.C. (2008). Stem Cells and Niches: Mechanisms That Promote Stem Cell Maintenance throughout Life. Cell.

[8] Plikus M.V., Wang X., Sinha S., Forte E., Thompson S.M., Herzog E.L., Driskell R.R., Rosenthal N., Biernaskie J., Horsley V. (2021). Fibroblasts: Origins, definitions, and functions in health and disease. Cell.

[9] Junker J.P., Sommar P., Skog M., Johnson H., Kratz G. (2009). Adipogenic, Chondrogenic and Osteogenic Differentiation of Clonally Derived Human Dermal Fibroblasts. Cells Tissues Organs.

[10] Rivera-Gonzalez G.C., Shook B.A., Andrae J., Holtrup B., Bollag K., Betsholtz C., Rodeheffer M.S., Horsley V. (2016). Skin Adipocyte Stem Cell Self-Renewal Is Regulated by a PDGFA/AKT-Signaling Axis. Cell Stem Cell.

[11] Ferrer R.A., Saalbach A., Grünwedel M., Lohmann N., Forstreuter I., Saupe S., Wandel E., Simon J.C., Franz S. (2017). Dermal Fibroblasts Promote Alternative Macrophage Activation Improving Impaired Wound Healing. J. Investig. Dermatol..

[12] Van Linthout S., Miteva K., Tschöpe C. (2014). Crosstalk between fibroblasts and inflammatory cells. Cardiovasc. Res..

[13] Nakaya M., Watari K., Tajima M., Nakaya T., Matsuda S., Ohara H., Nishihara H., Yamaguchi H., Hashimoto A., Nishida M. (2016). Cardiac myofibroblast engulfment of dead cells facilitates recovery after myocardial infarction. J. Clin. Investig..

[14] Jia Z., Wei Y., Zhang Y., Song K., Yuan J. (2024). Metabolic reprogramming and heterogeneity during the decidualization process of endometrial stromal cells. Cell Commun. Signal..

[15] Barragan F., Irwin J.C., Balayan S., Erikson D.W., Chen J.C., Houshdaran S., Piltonen T.T., Spitzer T.L., George A., Rabban J.T. (2016). Human Endometrial Fibroblasts Derived from Mesenchymal Progenitors Inherit Progesterone Resistance and Acquire an Inflammatory Phenotype in the Endometrial Niche in Endometriosis. Biol. Reprod..

[16] Ng S.-W., Norwitz G.A., Pavlicev M., Tilburgs T., Simón C., Norwitz E.R. (2020). Endometrial Decidualization: The Primary Driver of Pregnancy Health. Int. J. Mol. Sci..

[17] Gellersen B., Brosens J.J. (2014). Cyclic Decidualization of the Human Endometrium in Reproductive Health and Failure. Endocr. Rev..

[18] Kirkwood P.M., A Gibson D., Shaw I., Dobie R., Kelepouri O., Henderson N.C., Saunders P.T. (2022). Single-cell RNA sequencing and lineage tracing confirm mesenchyme to epithelial transformation (MET) contributes to repair of the endometrium at menstruation. eLife.

[19] Menkhorst E., So T., Rainczuk K., Barton S., Zhou W., Edgell T., Dimitriadis E. (2023). Endometrial stromal cell miR-19b-3p release is reduced during decidualization implying a role in decidual-trophoblast cross-talk. Front. Endocrinol..

[20] Guler Z., Roovers J.P. (2022). Role of Fibroblasts and Myofibroblasts on the Pathogenesis and Treatment of Pelvic Organ Prolapse. Biomolecules.

[21] Sahai E., Astsaturov I., Cukierman E., DeNardo D.G., Egeblad M., Evans R.M., Fearon D., Greten F.R., Hingorani S.R., Hunter T. (2020). A framework for advancing our understanding of cancer-associated fibroblasts. Nat. Rev. Cancer.

[22] Villegas-Pineda J.C., Ramírez-De-Arellano A., Bueno-Urquiza L.J., Lizarazo-Taborda M.d.R., Pereira-Suárez A.L. (2023). Cancer-associated fibroblasts in gynecological malignancies: Are they really allies of the enemy?. Front. Oncol..

[23] Lee Y.T., Tan Y.J., Falasca M., Oon C.E. (2020). Cancer-Associated Fibroblasts: Epigenetic Regulation and Therapeutic Intervention in Breast Cancer. Cancers.

[24] Muhl L., Genové G., Leptidis S., Liu J., He L., Mocci G., Sun Y., Gustafsson S., Buyandelger B., Chivukula I.V. (2020). Single-cell analysis uncovers fibroblast heterogeneity and criteria for fibroblast and mural cell identification and discrimination. Nat. Commun..

[25] Carr H.L., Turner J.D., Major T., Scheel-Toellner D., Filer A. (2020). New Developments in Transcriptomic Analysis of Synovial Tissue. Front. Med..

[26] Xia Y., Wang Y., Shan M., Hao Y., Liang Z. (2023). Decoding the molecular landscape of keloids: New insights from single-cell transcriptomics. Burn. Trauma.

[27] Zhang H., Zhang C., Zhang S. (2023). Single-Cell RNA Transcriptome of the Human Endometrium Reveals Epithelial Characterizations Associated with Recurrent Implantation Failure. Adv. Biol..

[28] Wang W., Vilella F., Alama P., Moreno I., Mignardi M., Isakova A., Pan W., Simon C., Quake S.R. (2020). Single-cell transcriptomic atlas of the human endometrium during the menstrual cycle. Nat. Med..

[29] Wu B., Li Y., Nie N., Shen X., Jiang W., Liu Y., Gong L., An C., Zhao K., Yao X. (2022). SFRP4+ stromal cell subpopulation with IGF1 signaling in human endometrial regeneration. Cell Discov..

[30] Garcia-Alonso L., Handfield L.-F., Roberts K., Nikolakopoulou K., Fernando R.C., Gardner L., Woodhams B., Arutyunyan A., Polanski K., Hoo R. (2021). Mapping the temporal and spatial dynamics of the human endometrium in vivo and in vitro. Nat. Genet..

[31] Yildiz S., Kinali M., Wei J.J., Milad M., Yin P., Adli M., Bulun S.E. (2023). Adenomyosis: Single-cell transcriptomic analysis reveals a paracrine mesenchymal–epithelial interaction involving the WNT/SFRP pathway. Fertil. Steril..

[32] Li H., Peng H., Hong W., Wei Y., Tian H., Huang X., Jia L., Zheng J., Duan T., He Q. (2022). Human Placental Endothelial Cell and Trophoblast Heterogeneity and Differentiation Revealed by Single-Cell RNA Sequencing. Cells.

[33] Nezhat C., Li A., Abed S., Balassiano E., Soliemannjad R., Nezhat A., Nezhat C.H., Nezhat F. (2016). Strong Association Between Endometriosis and Symptomatic Leiomyomas. JSLS J. Soc. Laparosc. Robot. Surg..

[34] Zhu S., Wang A., Xu W., Hu L., Sun J., Wang X. (2022). The heterogeneity of fibrosis and angiogenesis in endometriosis revealed by single-cell RNA-sequencing. Biochim. Biophys. Acta (BBA)-Mol. Basis Dis..

[35] Shin S., Chung Y., Moon S.W., Choi E.J., Kim M., Chung Y., Lee S.H. (2023). Single-cell profiling identifies distinct hormonal, immunologic, and inflammatory signatures of endometriosis-constituting cells. J. Pathol..

[36] Ma J., Zhang L., Zhan H., Mo Y., Ren Z., Shao A., Lin J. (2021). Single-cell transcriptomic analysis of endometriosis provides insights into fibroblast fates and immune cell heterogeneity. Cell Biosci..

[37] McKinnon B.D., Lukowski S.W., Mortlock S., Crawford J., Atluri S., Subramaniam S., Johnston R.L., Nirgianakis K., Tanaka K., Amoako A. (2022). Altered differentiation of endometrial mesenchymal stromal fibroblasts is associated with endometriosis susceptibility. Commun. Biol..

[38] Goad J., Rudolph J., Zandigohar M., Tae M., Dai Y., Wei J.-J., E Bulun S., Chakravarti D., Rajkovic A. (2022). Single-cell sequencing reveals novel cellular heterogeneity in uterine leiomyomas. Hum. Reprod..

[39] Xu J., Fang Y., Chen K., Li S., Tang S., Ren Y., Cen Y., Fei W., Zhang B., Shen Y. (2022). Single-Cell RNA Sequencing Reveals the Tissue Architecture in Human High-Grade Serous Ovarian Cancer. Clin. Cancer Res..

[40] Loret N., Vandamme N., De Coninck J., Taminau J., De Clercq K., Blancke G., Jonckheere S., Goossens S., Lemeire K., De Prijck S. (2022). Distinct Transcriptional Programs in Ascitic and Solid Cancer Cells Induce Different Responses to Chemotherapy in High-Grade Serous Ovarian Cancer. Mol. Cancer Res..

[41] Izar B., Tirosh I., Stover E.H., Wakiro I., Cuoco M.S., Alter I., Rodman C., Leeson R., Su M.-J., Shah P. (2020). A single-cell landscape of high-grade serous ovarian cancer. Nat. Med..

[42] Fang Y., Xiao X., Wang J., Dasari S., Pepin D., Nephew K.P., Zamarin D., Mitra A.K. (2024). Cancer associated fibroblasts serve as an ovarian cancer stem cell niche through noncanonical Wnt5a signaling. NPJ Precis. Oncol..

[43] Hu Y., Recouvreux M.S., Haro M., Taylan E., Taylor-Harding B., Walts A.E., Karlan B.Y., Orsulic S. (2024). INHBA(+) cancer-associated fibroblasts generate an immunosuppressive tumor microenvironment in ovarian cancer. NPJ Precis. Oncol..

[44] Xu J., Lu W., Wei X., Zhang B., Yang H., Tu M., Chen X., Wu S., Guo T. (2024). Single-cell transcriptomics reveals the aggressive landscape of high-grade serous carcinoma and therapeutic targets in tumor microenvironment. Cancer Lett..

[45] Denisenko E., de Kock L., Tan A., Beasley A.B., Beilin M., Jones M.E., Hou R., Muirí D., Bilic S., Mohan G.R.K.A. (2024). Spatial transcriptomics reveals discrete tumour microenvironments and autocrine loops within ovarian cancer subclones. Nat. Commun..

[46] Ma C., Yang C., Peng A., Sun T., Ji X., Mi J., Wei L., Shen S., Feng Q. (2023). Pan-cancer spatially resolved single-cell analysis reveals the crosstalk between cancer-associated fibroblasts and tumor microenvironment. Mol. Cancer.

[47] Li Y., Wang W., Wang D., Zhang L., Wang X., He J., Cao L., Li K., Xie H. (2022). Single-Cell Sequencing of Malignant Ascites Reveals Transcriptomic Remodeling of the Tumor Microenvironment during the Progression of Epithelial Ovarian Cancer. Genes.

[48] Kan T., Zhang S., Zhou S., Zhang Y., Zhao Y., Gao Y., Zhang T., Gao F., Wang X., Zhao L. (2022). Single-cell RNA-seq recognized the initiator of epithelial ovarian cancer recurrence. Oncogene.

[49] Shih A.J., Menzin A., Whyte J., Lovecchio J., Liew A., Khalili H., Bhuiya T., Gregersen P.K., Lee A.T. (2018). Identification of grade and origin specific cell populations in serous epithelial ovarian cancer by single cell RNA-seq. PLoS ONE.

[50] Mori Y., Okimoto Y., Sakai H., Kanda Y., Ohata H., Shiokawa D., Suzuki M., Yoshida H., Ueda H., Sekizuka T. (2024). Targeting PDGF signaling of cancer-associated fibroblasts blocks feedback activation of HIF-1α and tumor progression of clear cell ovarian cancer. Cell Rep. Med..

[51] Ye Y., Zhang S., Jiang Y., Huang Y., Wang G., Zhang M., Gui Z., Wu Y., Bian G., Li P. (2023). Identification of a cancer associated fibroblasts-related index to predict prognosis and immune landscape in ovarian cancer. Sci. Rep..

[52] Ye C.-J., Zhan Y., Yang R., Li Y., Dong R. (2020). Single-cell transcriptional profiling identifies a cluster of potential metastasis-associated *UBE2C*+ cells in immature ovarian teratoma. Biochem. Biophys. Res. Commun..

[53] Yu Z., Zhang J., Zhang Q., Wei S., Shi R., Zhao R., An L., Grose R., Feng D., Wang H. (2022). Single-cell sequencing reveals the heterogeneity and intratumoral crosstalk in human endometrial cancer. Cell Prolif..

[54] Dong C., Zhao L., Liu X., Dang L., Zhang X. (2024). Single-cell analysis reveals landscape of endometrial cancer response to estrogen and identification of early diagnostic markers. PLoS ONE.

[55] Szabo P.M., Vajdi A., Kumar N., Tolstorukov M.Y., Chen B.J., Edwards R., Ligon K.L., Chasalow S.D., Chow K.-H., Shetty A. (2023). Cancer-associated fibroblasts are the main contributors to epithelial-to-mesenchymal signatures in the tumor microenvironment. Sci. Rep..

[56] Li C., Wu H., Guo L., Liu D., Yang S., Li S., Hua K. (2022). Single-cell transcriptomics reveals cellular heterogeneity and molecular stratification of cervical cancer. Commun. Biol..

[57] Li C., Liu D., Yang S., Hua K. (2022). Integrated single-cell transcriptome analysis of the tumor ecosystems underlying cervical cancer metastasis. Front. Immunol..

[58] Lin S., Sun Y., Cao C., Zhu Z., Xu Y., Liu B., Hu B., Peng T., Zhi W., Xu M. (2023). Single-nucleus RNA sequencing reveals heterogenous microenvironments and specific drug response between cervical squamous cell carcinoma and adenocarcinoma. EBioMedicine.

[59] Qiu J., Qu X., Wang Y., Guo C., Lv B., Jiang Q., Su W., Wang L., Hua K. (2023). Single-Cell Landscape Highlights Heterogenous Microenvironment, Novel Immune Reaction Patterns, Potential Biomarkers and Unique Therapeutic Strategies of Cervical Squamous Carcinoma, Human Papillomavirus-Associated (HPVA) and Non-HPVA Adenocarcinoma. Adv. Sci..

[60] Li C., Liu D., Zhao Y., Ding Y., Hua K. (2023). Diverse intratumoral heterogeneity and immune microenvironment of two HPV-related cervical cancer types revealed by single-cell RNA sequencing. J. Med. Virol..

[61] Wen S., Lv X., Li P., Li J., Qin D. (2023). Analysis of cancer-associated fibroblasts in cervical cancer by single-cell RNA sequencing. Aging.

[62] Zhang L., Ma J., Zhou D., Zhou J., Hu B., Ma X., Tang J., Bai Y., Chen H., Jing Y. (2023). Single-Nucleus Transcriptome Profiling of Locally Advanced Cervical Squamous Cell Cancer Identifies Neural-Like Progenitor Program Associated with the Efficacy of Radiotherapy. Adv. Sci..

[63] Wang Y., Xu M., Yao Y., Li Y., Zhang S., Fu Y., Wang X. (2024). Extracellular cancer-associated fibroblasts: A novel subgroup in the cervical cancer microenvironment that exhibits tumor-promoting roles and prognosis biomarker functions. Oncol. Lett..

[64] Ou Z., Lin S., Qiu J., Ding W., Ren P., Chen D., Wang J., Tong Y., Wu D., Chen A. (2022). Single-Nucleus RNA Sequencing and Spatial Transcriptomics Reveal the Immunological Microenvironment of Cervical Squamous Cell Carcinoma. Adv. Sci..

[65] Liu C., Zhang M., Yan X., Ni Y., Gong Y., Wang C., Zhang X., Wan L., Yang H., Ge C. (2023). Single-cell dissection of cellular and molecular features underlying human cervical squamous cell carcinoma initiation and progression. Sci. Adv..

[66] Fan J., Lu F., Qin T., Peng W., Zhuang X., Li Y., Hou X., Fang Z., Yang Y., Guo E. (2023). Multiomic analysis of cervical squamous cell carcinoma identifies cellular ecosystems with biological and clinical relevance. Nat. Genet..

[67] Erikson D.W., Barragan F., Piltonen T.T., Chen J.C., Balayan S., Irwin J.C., Giudice L.C. (2017). Stromal fibroblasts from perimenopausal endometrium exhibit a different transcriptome than those from the premenopausal endometrium†. Biol. Reprod..

[68] Warren L.A., Shih A., Renteira S.M., Seckin T., Blau B., Simpfendorfer K., Lee A., Metz C.N., Gregersen P.K. (2018). Analysis of menstrual effluent: Diagnostic potential for endometriosis. Mol. Med..

[69] Koumas L., King A.E., Critchley H.O., Kelly R.W., Phipps R.P. (2001). Fibroblast Heterogeneity: Existence of Functionally Distinct Thy 1+ and Thy 1− Human Female Reproductive Tract Fibroblasts. Am. J. Pathol..

[70] Piltonen T.T., Chen J., Erikson D.W., Spitzer T.L., Barragan F., Rabban J.T., Huddleston H., Irwin J.C., Giudice L.C. (2013). Mesenchymal stem/progenitors and other endometrial cell types from women with polycystic ovary syndrome (PCOS) display inflammatory and oncogenic potential. J. Clin. Endocrinol. Metab..

[71] Holdsworth-Carson S.J., Zaitseva M., Vollenhoven B.J., Rogers P.A. (2013). Clonality of smooth muscle and fibroblast cell populations isolated from human fibroid and myometrial tissues. Mol. Hum. Reprod..

[72] Holdsworth-Carson S.J., Zaitseva M., E Girling J., Vollenhoven B.J., Rogers P.A.W. (2014). Common fibroid-associated genes are differentially expressed in phenotypically dissimilar cell populations isolated from within human fibroids and myometrium. Reproduction.

[73] Sima Y., Li J., Xiao C., Xu L., Wang L., Chen Y. (2022). *CD*106/VCAM-1 distinguishes a fibroblast subpopulation with high colony-forming capacity and distinct protein expression from the uterosacral ligament. Ann. Transl. Med..

[74] Riddell M., Winkler-Lowen B., Chakrabarti S., Dunk C., Davidge S., Guilbert L. (2012). The characterization of fibrocyte-like cells: A novel fibroblastic cell of the placenta. Placenta.

[75] Boss A.L., Damani T., Wickman T.J., Chamley L.W., James J.L., Brooks A.E. (2022). Full spectrum flow cytometry reveals mesenchymal heterogeneity in first trimester placentae and phenotypic convergence in culture, providing insight into the origins of placental mesenchymal stromal cells. eLife.

[76] Chen D., Smith L.R., Khandekar G., Patel P., Yu C.K., Zhang K., Chen C.S., Han L., Wells R.G. (2020). Distinct effects of different matrix proteoglycans on collagen fibrillogenesis and cell-mediated collagen reorganization. Sci. Rep..

[77] Merline R., Schaefer R.M., Schaefer L. (2009). The matricellular functions of small leucine-rich proteoglycans (SLRPs). J. Cell Commun. Signal..

[78] Ostrowska-Podhorodecka Z., McCulloch C.A. (2021). Vimentin regulates the assembly and function of matrix adhesions. Wound Repair Regen..

[79] Arrindell J., Desnues B. (2023). Vimentin: From a cytoskeletal protein to a critical modulator of immune response and a target for infection. Front. Immunol..

[80] Smith B.N., Bhowmick N.A. (2016). Role of EMT in Metastasis and Therapy Resistance. J. Clin. Med..

[81] Yang J., Zhan X.-Z., Malola J., Li Z.-Y., Pawar J.S., Zhang H.-T., Zha Z.-G. (2020). The multiple roles of Thy-1 in cell differentiation and regeneration. Differentiation.

[82] Demoulin J.-B., Essaghir A. (2014). PDGF receptor signaling networks in normal and cancer cells. Cytokine Growth Factor Rev..

[83] Fitzgerald A.A., Weiner L.M. (2020). The role of fibroblast activation protein in health and malignancy. Cancer Metastasis Rev..

[84] Olazaba O.L., Farrow J., Monkkonen T. (2024). Fibroblast heterogeneity and functions: Insights from single-cell sequencing in wound healing, breast cancer, ovarian cancer and melanoma. Front. Genet..

[85] Lavie D., Ben-Shmuel A., Erez N., Scherz-Shouval R. (2022). Cancer-associated fibroblasts in the single-cell era. Nat. Cancer.

[86] Cooke P.S., Mesa A.M., Sirohi V.K., Levin E.R. (2020). Role of nuclear and membrane estrogen signaling pathways in the male and female reproductive tract. Differentiation.

[87] Hewitt S.C., Wu S.-P., Wang T., Ray M., Brolinson M., Young S.L., E Spencer T., DeCherney A., DeMayo F.J. (2022). The Estrogen Receptor α Cistrome in Human Endometrium and Epithelial Organoids. Endocrinology.

[88] Nalivaeva N.N., Zhuravin I.A., Turner A.J. (2020). Neprilysin expression and functions in development, ageing and disease. Mech. Ageing Dev..

[89] Donovan J., Shiwen X., Norman J., Abraham D. (2013). Platelet-derived growth factor alpha and beta receptors have overlapping functional activities towards fibroblasts. Fibrogenesis Tissue Repair.

[90] Lala T., Hall R.A. (2022). Adhesion G protein-coupled receptors: Structure, signaling, physiology, and pathophysiology. Physiol. Rev..

[91] Atanasova V.S., Russell R.J., Webster T.G., Cao Q., Agarwal P., Lim Y.Z., Krishnan S., Fuentes I., Guttmann-Gruber C., McGrath J.A. (2019). Thrombospondin-1 is a major activator of TGF-beta signaling in recessive dystrophic epidermolysis bullosa fibroblasts. J. Investig. Dermatol..

[92] Hoter A., El-Sabban M.E., Naim H.Y. (2018). The HSP90 Family: Structure, Regulation, Function, and Implications in Health and Disease. Int. J. Mol. Sci..

[93] Monnin N., Fattet A.J., Koscinski I. (2023). Endometriosis: Update of Pathophysiology, (Epi) Genetic and Environmental Involvement. Biomedicines.

[94] Moore A.B., Yu L., Swartz C.D., Zheng X., Wang L., Castro L., E Kissling G., Walmer D.K., Robboy S.J., Dixon D. (2010). Human uterine leiomyoma-derived fibroblasts stimulate uterine leiomyoma cell proliferation and collagen type I production, and activate RTKs and TGF beta receptor signaling in coculture. Cell Commun. Signal..

[95] Li J., Zhou C., Gao X., Tan T., Zhang M., Li Y., Chen H., Wang R., Wang B., Liu J. (2024). S100A10 promotes cancer metastasis via recruitment of MDSCs within the lungs. OncoImmunology.

[96] Li H., Korcari A., Ciufo D., Mendias C.L., Rodeo S.A., Buckley M.R., Loiselle A.E., Pitt G.S., Cao C. (2023). Increased Ca^2+^ signaling through Ca_V_1.2 induces tendon hypertrophy with increased collagen fibrillogenesis and biomechanical properties. FASEB J..

[97] Ohashi K., Li T.-S., Miura S., Hasegawa Y., Miura K. (2022). Biological Differences Between Ovarian Cancer-associated Fibroblasts and Contralateral Normal Ovary-derived Mesenchymal Stem Cells. Anticancer. Res..

[98] Zhang M., Chen Z., Wang Y., Zhao H., Du Y. (2022). The Role of Cancer-Associated Fibroblasts in Ovarian Cancer. Cancers.

[99] Veneziani A.C., Gonzalez-Ochoa E., Alqaisi H., Madariaga A., Bhat G., Rouzbahman M., Sneha S., Oza A.M. (2023). Heterogeneity and treatment landscape of ovarian carcinoma. Nat. Rev. Clin. Oncol..

[100] Lengyel E. (2010). Ovarian Cancer Development and Metastasis. Am. J. Pathol..

[101] Chen Y., Liang Z., Lai M. (2024). Targeting the devil: Strategies against cancer-associated fibroblasts in colorectal cancer. Transl. Res..

[102] Wright K., Ly T., Kriet M., Czirok A., Thomas S.M. (2023). Cancer-Associated Fibroblasts: Master Tumor Microenvironment Modifiers. Cancers.

[103] Mao X., Xu J., Wang W., Liang C., Hua J., Liu J., Zhang B., Meng Q., Yu X., Shi S. (2021). Crosstalk between cancer-associated fibroblasts and immune cells in the tumor microenvironment: New findings and future perspectives. Mol. Cancer.

[104] Zhang S., Gong T.-T., Liu F.-H., Jiang Y.-T., Sun H., Ma X.-X., Zhao Y.-H., Wu Q.-J. (2019). Global, Regional, and National Burden of Endometrial Cancer, 1990–2017: Results from the Global Burden of Disease Study, 2017. Front. Oncol..

[105] Xu Q., Wang Y., Zhu J., Zhao Y., Lin Y. (2020). Molecular characterization of GTP binding protein overexpressed in skeletal muscle (GEM) and its role in promoting adipogenesis in goat intramuscular preadipocytes. Anim. Biotechnol..

[106] Wang Y., Wang K., Zhang H., Jia X., Li X., Sun S., Sun D. (2023). Cell death-related biomarker SLC2A1 has a significant role in prognosis prediction and immunotherapy efficacy evaluation in pan-cancer. Front. Genet..

[107] Zheng H., Long G., Zheng Y., Yang X., Cai W., He S., Qin X., Liao H. (2022). Glycolysis-Related SLC2A1 Is a Potential Pan-Cancer Biomarker for Prognosis and Immunotherapy. Cancers.

[108] Cui Y., Li J., Zhang P., Yin D., Wang Z., Dai J., Wang W., Zhang E., Guo R. (2023). B4GALT1 promotes immune escape by regulating the expression of PD-L1 at multiple levels in lung adenocarcinoma. J. Exp. Clin. Cancer Res..

[109] Buskwofie A., David-West G., Clare C.A. (2020). A Review of Cervical Cancer: Incidence and Disparities. J. Natl. Med. Assoc..

[110] Chen L., Alabdullah M., Mahnke K. (2023). Adenosine, bridging chronic inflammation and tumor growth. Front. Immunol..

[111] Ollauri-Ibáñez C., Ayuso-Íñigo B., Pericacho M. (2021). Hot and Cold Tumors: Is Endoglin (CD105) a Potential Target for Vessel Normalization?. Cancers.

[112] Kong D.-H., Kim Y.K., Kim M.R., Jang J.H., Lee S. (2018). Emerging Roles of Vascular Cell Adhesion Molecule-1 (VCAM-1) in Immunological Disorders and Cancer. Int. J. Mol. Sci..

[113] Tabib T., Morse C., Wang T., Chen W., Lafyatis R. (2017). SFRP2/DPP4 and FMO1/LSP1 Define Major Fibroblast Populations in Human Skin. J. Investig. Dermatol..

